# Recent Advances in Structural Optimization and Surface Modification on Current Collectors for High-Performance Zinc Anode: Principles, Strategies, and Challenges

**DOI:** 10.1007/s40820-023-01177-4

**Published:** 2023-08-31

**Authors:** Yuxin Gong, Bo Wang, Huaizheng Ren, Deyu Li, Dianlong Wang, Huakun Liu, Shixue Dou

**Affiliations:** 1 MIIT Key Laboratory of Critical Materials Technology for New Energy Conversion and Storage, State Key Laboratory of Urban Water Resource and Environment, School of Chemistry and Chemical Engineering, Harbin Institute of Technology, Harbin, 150001 Heilongjiang People’s Republic of China; 2 Key Laboratory of Advanced Energy Materials Chemistry (Ministry of Education), College of Chemistry, Nankai University, Tianjin, 300071 People’s Republic of China; 3 Institute of Energy Material Science, University of Shanghai for Science and Technology, Shanghai, 200093 People’s Republic of China

**Keywords:** Zinc anodes, Current collectors, Surface modification, Structural design, Crystal facet orientation

## Abstract

The last several years have witnessed the prosperous development of zinc-ion batteries (ZIBs), which are considered as a promising competitor of energy storage systems thanks to their low cost and high safety. However, the reversibility and availability of this system are blighted by problems such as uncontrollable dendritic growth, hydrogen evolution, and corrosion passivation on anode side. A functionally and structurally well-designed anode current collectors (CCs) is believed as a viable solution for those problems, with a lack of summarization according to its working mechanisms. Herein, this review focuses on the challenges of zinc anode and the mechanisms of modified anode CCs, which can be divided into zincophilic modification, structural design, and steering the preferred crystal facet orientation. The possible prospects and directions on zinc anode research and design are proposed at the end to hopefully promote the practical application of ZIBs.

**Supplementary Information:**

The online version contains supplementary material available at 10.1007/s40820-023-01177-4.

## Highlights

- The mechanisms of the surface modification and structure design of zinc anode current collectors were summarized.
- The recent advances of high-performance zinc anode current collectors were reviewed and categorized according to their working mechanisms.
- The possible prospects and directions of zinc anode research were discussed.

**Supplementary Information:**

The online version contains supplementary material available at 10.1007/s40820-023-01177-4.

## Graphical abstract

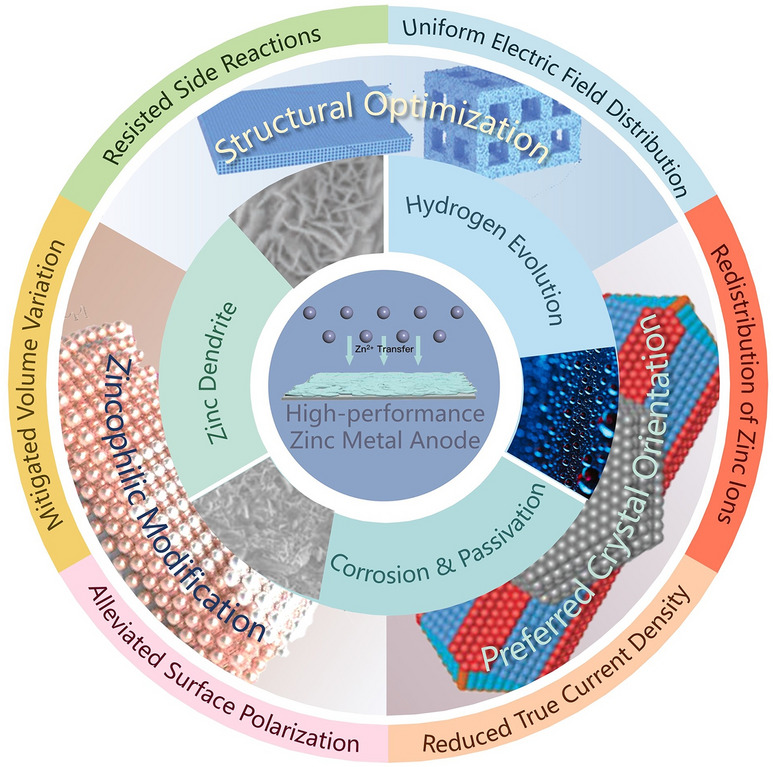

## Introduction

The imminent menace of global warming and the exhaustion of fossil fuel have called for the prosperous development of energy conversion and storage, and lithium-ion batteries, as a representative, have been widely used in consumer electronics and electric vehicles thanks to their high energy density and long cycle life [1–3]. However, the sustainable progression of Li-ion batteries is suffering from lithium resource shortage and safety problems, which prompt people to develop alternatives [4, 5]. The aqueous metal batteries, compared to their organic-electrolyte-based peers such as normal Li-ion and Na-ion batteries, are believed to be more promising owing to their higher ionic conductivity, lower cost, lower assembling difficulty, and higher intrinsic safety [6–8]. Among them, the zinc metal anode has a high theoretical capacity (820 mAh g^−1^ and 5855 mAh cm^−3^), low redox potential (− 0.76 V vs. standard hydrogen electrode), high hydrogen evolution overpotential, and excellent safety [9, 10]. Hence, the aqueous Zn-ion batteries show great potential for application in large-scale energy storage systems [11].

As an anode, zinc metal has a rich history of use in batteries, dating back to the eighteenth-century voltaic pile [12]. Zinc-ion batteries (ZIBs) are a type of zinc-ion battery that uses mildly acidic aqueous electrolytes, first introduced by Yamamoto et al*.* in 1988 as the rechargeable Zn|ZnSO_4_|MnO_2_ battery system [13]. Unfortunately, this battery system was plagued by uncontrollable zinc dendrite growth [14], the structural failure of cathodes [15, 16], and electrolyte decomposition [17, 18], all of which prevented it from being truly operational. With the in-depth research on the cathodes of ZIBs, the problems which limited the capacity of ZIBs, have been alleviated [19–21]. However, the problem that currently hinders the widespread application of ZIBs is the growth of dendrites and side reactions on the anode side of zinc batteries, which may lead to short circuits and the increase in the inner pressure [22]. The meticulous designation of the surface and structure of anode current collectors (CCs) has been thought of as one of the viable strategies to diminish the impact of anode problems. Although many methods have been proposed, there are few summaries of the variety of these methods, even fewer of them are systematically classified according to their mechanisms.

This review will be structured as follows: First, we will examine the challenges encountered by researchers in their studies, which can be divided into two main categories: dendrite formation and side reactions. Next, we will discuss recent design strategies for anode CCs, organized by mechanism and subdivided into three main categories: zincophilic modification, structural optimization, and crystal orientation preferred coating layer. Finally, we will provide a summary of these design strategies and offer some prospects for future research.

## Issues and Challenges

Zinc anodes, similar to their lithium and sodium counterparts, are not immune to the challenges arising from the inhomogeneity of the anode surface and the reactivity of the metals [23]. The formation of dendrites, solvent-induced parasitic reactions, and gas production—problems that have plagued lithium and sodium anodes for decades—have also been observed in zinc systems. These issues can severely impede the practical application of zinc anodes [24]. In the following section, we will describe the kinetic process of zinc deposition and the mechanisms of zinc dendrite and side reactions.

**Fig. 1:**
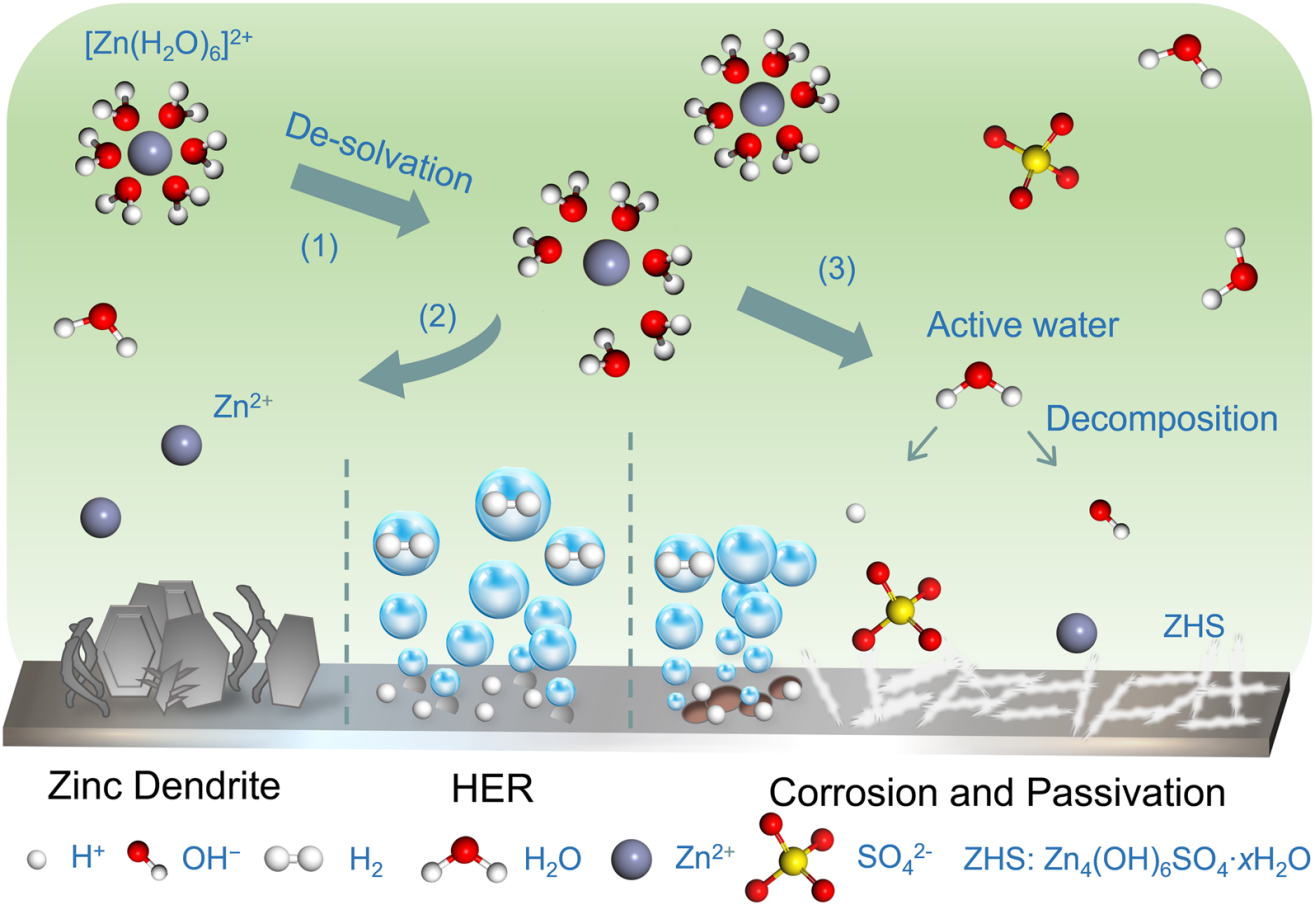
Schematic illustration showing the main challenges in zinc anodes

Zinc ions (Zn^2+^) are known for their small ionic radius and strong solvation due to their double valence [25]. As a result, they are often found in electrolytes as solvent-coordinated cations [26]. One common example of a mildly acidic electrolyte is 2 mol L^−1^ ZnSO_4_, where Zn^2+^ exists in the form of hydrated zinc ion [Zn(H_2_O)_6_]^2+^. In terms of electrochemical kinetic theory [27], the deposition of Zn ions on the anode side occurs in a series of steps, starting with mass transfer, followed by surface transformation, and finally nucleation/electron transfer. When [Zn(H_2_O)_6_]^2+^ diffuses and adsorbs to the active site on the anode surface, it tends to undergo a surface transformation process: shedding some of the ligand water to form a more easily discharging form, [Zn(H_2_O)_6-*n*_]^2+^, with *n* possibly varying according to $C_{{{\text{Zn}}^{2 + } }}$ in the electrolyte (process (1) in Fig. 1). A high activation energy is often required for the removal of ligands, and therefore, this process tends to take place at sites with higher energy on the electrode surface. From the kinetic point of view, this solventized structure increases the overpotential for Zn^2+^ nucleation and is detrimental to the uniform deposition of Zn^2+^ [28, 29]. Subsequently, the Zn^2+^ in the form of [Zn(H_2_O)_6-*n*_]^2+^ gains electrons and further sheds all ligands to form adsorbed zinc (Zn_ad_) on the electrode surface (process (2) in Fig. 1) [30, 31].

Zn_ad_ can either incorporate into the preexisting lattice and grow epitaxially along the crystal plane or create new nuclei at the active site [32]. As newly formed grains grow epitaxially along randomly orientated crystalline planes, irregular perpendicular sheets of grains can be produced, which may penetrate the separators as the grains continue to grow [33].

During the deposition process, the application of a potent electronegative electric field on the anode surface facilitates the rapid but uneven absorption and deposition of numerous cations, leading to the formation of dendrites. According to the “Sand model” [34], the growth of metal dendrites in dilute solutions initiates after a certain duration of time, denoted as *τ*. This time is in accordance with Eq. (1) [32, 35, 36]:

1 $$\tau = \pi D_{{{\text{Zn}}^{2 + } }} \frac{{\mu_{a} \left[ {eC_{{0,{\text{Zn}}^{2 + } }} \left( {1 + \frac{{\mu_{{{\text{Zn}}^{2 + } }} }}{{\mu_{a} }}} \right)} \right]^{2} }}{{2i^{2} }}$$

where the $C_{{{\text{0,Zn}}^{{2 + }} }}$, $\mu_{{\text{a}}},$ and *μ*_Zn_^2+^ represent the initial concentration in electrolyte, the mobility of anions, and Zn^2+^, respectively. According to the Sand model, the $D_{{{\text{Zn}}^{{2 + }} }}$ and *i* are critical to the formation of zinc dendrite; however, with a much smaller diffusion coefficient compared to Zn^2+^, the diffusion rate of [Zn(H_2_O)_6_]^2+^ is seriously affected by its solvation sheath [37]. Furthermore, this also leads to preferential adsorption of Zn^2+^ on the protrusions that are in closer proximity to the bulk electrolyte, resulting in a non-uniform distribution of Zn^2+^ on the surface of the anode [38, 39].

To summarize, in the electrodeposition model of Zn^2+^, the solvation sheath of Zn^2+^ and the surface condition on the anode have a decisive effect on the homogeneity of the deposition morphology of Zn^2+^. The effects of these factors on the formation of zinc dendrites and side reactions on the anode surface will be described in the following sections.

### Growth of Zinc Dendrites

Nowadays, the zinc dendrite growth on the anode surface is believed to be one of the major challenges for the development of high-performance ZIBs [40, 41]. Compared to the lithium and sodium dendrites, the safety problems induced by zinc dendrite are less severe, thanks to the inflammable nature of aqueous electrolyte and the relatively stable chemical characteristics of zinc metal. With a higher Young’s module (*E*_Zn_ ≈ 108 GPa [10]; *E*_Na_ ≈ 10 GPa [42]; *E*_Li_ ≈ 5 GPa [43]) and grains size, however, zinc dendrite penetration could be more fatal to the cycling life of ZIBs.

The formation of zinc dendrites during zinc deposition is generally attributed to non-uniform Zn ion diffusion upon the anode surface and uneven surface electric field distribution [44–47]. Thus, the flatness of market-sourced zinc foils in research applications can significantly impact the uniformity of ion distribution. This is due to the presence of surface protrusions on the foil that can have detrimental effects, leading to a decrease in uniformity [48, 49]. As a result, some cusps inevitably appear during zinc deposition. These cusps create a stronger electric field, and their electric field distribution determines the preferential accumulation and deposition of Zn^2+^ on cusps, ultimately leading to the formation of zinc dendrites, known as the “tip effect” [39]. Therefore, the dendrites will continue to grow during operation [29, 50], until sharp and stubborn zinc dendrites eventually penetrate the glass fiber separators, resulting in short-circuiting and failure of the ZIBs. Even if the batteries can service despite the existence of rampant dendrites, these dendrites may lose contact with the anode after several cycles of zinc deposition/dissolution and become “dead zinc,” accompanied by significant mass loss of active zinc and reduction in the Coulombic efficiency (CE) [51, 52].

### Side Reactions on Anode Side

Just that the existence of active water molecules in the electrolyte, a variety of side reactions may occur with or without external current, which can be mainly divided into hydrogen evolution reaction, insoluble by-products, and self-corrosion.

#### Hydrogen Evolution Reaction (HER)

Within the mildly acidic or alkaline electrolytes common to ZIBs, an important competing reaction for zinc deposition ($\varphi_{{{\text{Zn}}^{{2 + }} {\text{/Zn}}}} = - {0}{{.763}}\;{\text{V}}\;{\text{vs}}. \;{\text{SHE}}$) during the charging process of the anode is the hydrogen evolution reaction (HER) on the anode surface ($\varphi_{{{\text{H}}^{ + } {\text{/H}}_{{2}} }} = 0 \;{\text{V}}\;{\text{vs}}. \;{\text{SHE}}$) (process (3) in Fig. 1) [53]. Reactions can be different according to the pH of electrolytes. In mildly acidic or neutral electrolytes, according to the Nernst Eq. (2), the evolution potential $\varphi_{{{\text{H}}^{ + } {\text{/H}}_{{2}} }}$ is higher than the electrodeposition potential $\varphi_{{{\text{Zn}}^{2 + } /{\text{Zn}}}}$, the reaction occurs by Eq. (3). In the sealed ZIBs system, the hydrogen produced by HER cannot be consumed by the electrode reaction, and the gradual gas accumulation on the anode surface reduces the interface contact area, eventually causing deformation, failure, and explosion [46, 54].

2 $$\varphi_{{{\text{H}}^{ + } {\text{/H}}_{{2}} }} = \frac{{{\text{RT}}}}{{{2}F}}{\text{ln}}\left( {\frac{{C\left( {{\text{H}}^{ + } } \right)^{{2}} }}{{\frac{{p\left( {{\text{H}}_{{2}} } \right)}}{{p^{\emptyset } }}}}} \right){ }$$

3 $${2\text{H}}^{ + } \left( {{\text{aq}}} \right){ + 2}e^{ - } \to {\text{H}}_{{2}} \left( {\text{g}} \right)$$

Furthermore, HER reduces the CE of the battery, resulting in a decreased life span due to the consumption of active water molecules and gradual electrolyte drying [55]. The consumption of H^+^ in acidic electrolytes and the formation of OH^−^ in alkaline electrolytes both raise the pH; thus, some insoluble by-products containing Zn^2+^ may also generate, which causes passivation of zinc anode surface, e.g., Zn_4_(OH)_6_SO_4_·*x*H_2_O (ZHS) in 2 mol L^−1^ ZnSO_4_ electrolyte [56].

#### Corrosion and Passivation

In spite of the relatively low reaction activity between zinc and water, the corrosion reactions in both alkaline and acidic electrolyte are believed as a Gordian knot, which constantly cause the self-discharge of ZIBs. In alkaline electrolytes, the chemical corrosion reaction is as Eq. (4) [57]:

4 $${\text{Zn}}^{{2 + }} {+2\text{OH}}^{ - } {+ 2\text{ H}}_{{2}} {\text{O}} \to {\text{Zn(OH)}}_{{4}}^{{{2} - }} {+\text{ H}}_{{2}}$$

In neutral or mildly acidic electrolytes, electrochemical corrosion is observed as a more dominant side reaction compared to chemical corrosion [44]. This phenomenon is particularly pronounced in the case of zinc powder anodes with large surface areas, as noted by Li et al. [58] in their investigation of the shelf aging and cyclic aging of zinc powder anodes in pouch cells. Even in the absence of external current, the electrochemical corrosion reaction consumes a significant amount of active zinc powder during shelf aging caused by the galvanic corrosion microcells, denoted as Zn|electrolyte|substrate whose electrode reactions shown by Eqs. (5) and (6). Macroscopically, this is evidenced by a substantial corrosion microcurrent and noticeable bulging of the pouch cell due to hydrogen accumulation. It is proved that the rate of this electrochemical corrosion reaction is much higher than the chemical corrosion reaction in mildly acidic electrolytes [58].

5 $${\text{Micro}}\;{\text{anodes}}\;{\text{on}}\;{\text{zinc}}\;{\text{surface}}{:}{\text{Zn}} - {2}e^{ - } \to {\text{Zn}}^{{2 + }}$$

6 $${\text{Micro}}\;{\text{cathodes}}\;{\text{on}}\;{\text{substrates}}{:} {2\text{H}}^{ + } \left( {{\text{aq}}} \right){ + 2}e^{ - } \to {\text{H}}_{{2}} \left( {\text{g}} \right)$$

The by-products of electrochemical corrosion vary depending on the anions in electrolytes. For instance, when using ZnSO_4_ electrolyte, the reaction is represented by Eq. (7) [59]:

7 $${4\text{Zn}}^{{2 + }} {\text{ + SO}}_{{4}}^{{{2} - }} {+6\text{ OH}}^{ - } { + }x{\text{H}}_{{2}} {\text{O}} \to {\text{Zn}}_{{4}} \left( {{\text{OH}}} \right)_{{6}} {\text{SO}}_{{4}} \cdot x{\text{H}}_{{2}} {\text{O}}\;{\text{(ZHS)}}$$

The problem of spontaneous zinc corrosion directly results in battery failure and exacerbates the difficulty of achieving optimal battery performance due to the by-products it generates. As highlighted in Sect. 2.2.1, in addition to the significant consumption of active material, the formation of hydrogen diminishes the CE, elevates its internal pressure, and ultimately leads to its failure [60]. Furthermore, the poorly conductive by-products tend to accumulate on the surface of the zinc anode, impeding the transfer of Zn^2+^ and electrons to the electrode/electrolyte interface [61]. This deteriorates the availability of active sites for surface zinc deposition/dissolution, thereby reducing the utilization of the zinc anode's active material and causing an increase in its irreversibility. All of the above increase the polarization of Zn^2+^ deposition, further reducing the homogeneity and reversibility of Zn^2+^ deposition/dissolution processes [31].

To guarantee the service life, similar self-corrosion reactions should be avoided as far as possible.

### Interaction Between the Dendrite Formation and Side Reactions

The problems of dendrite formation and side reactions are major factors contributing to the failure of ZIBs by depleting the active zinc and disrupting the battery's operation. These problems are not independent, as the formation of zinc dendrites significantly increases the surface area of the anode, creating more active sites for side reactions to take place. Moreover, the accumulation of bubbles from the HER at the anode/electrolyte interface and the formation of alkaline zinc salts due to pH changes degrade the interfacial contact and conductivity, exacerbating the dendrite problem and deteriorating the reversibility of zinc deposition/dissolution reactions. Therefore, it is crucial to address the dendrite and side reaction problems simultaneously by developing a comprehensive strategy that tackles these problems in an integrated manner [44].

## Strategies to the CC Modification

**Fig. 2:**
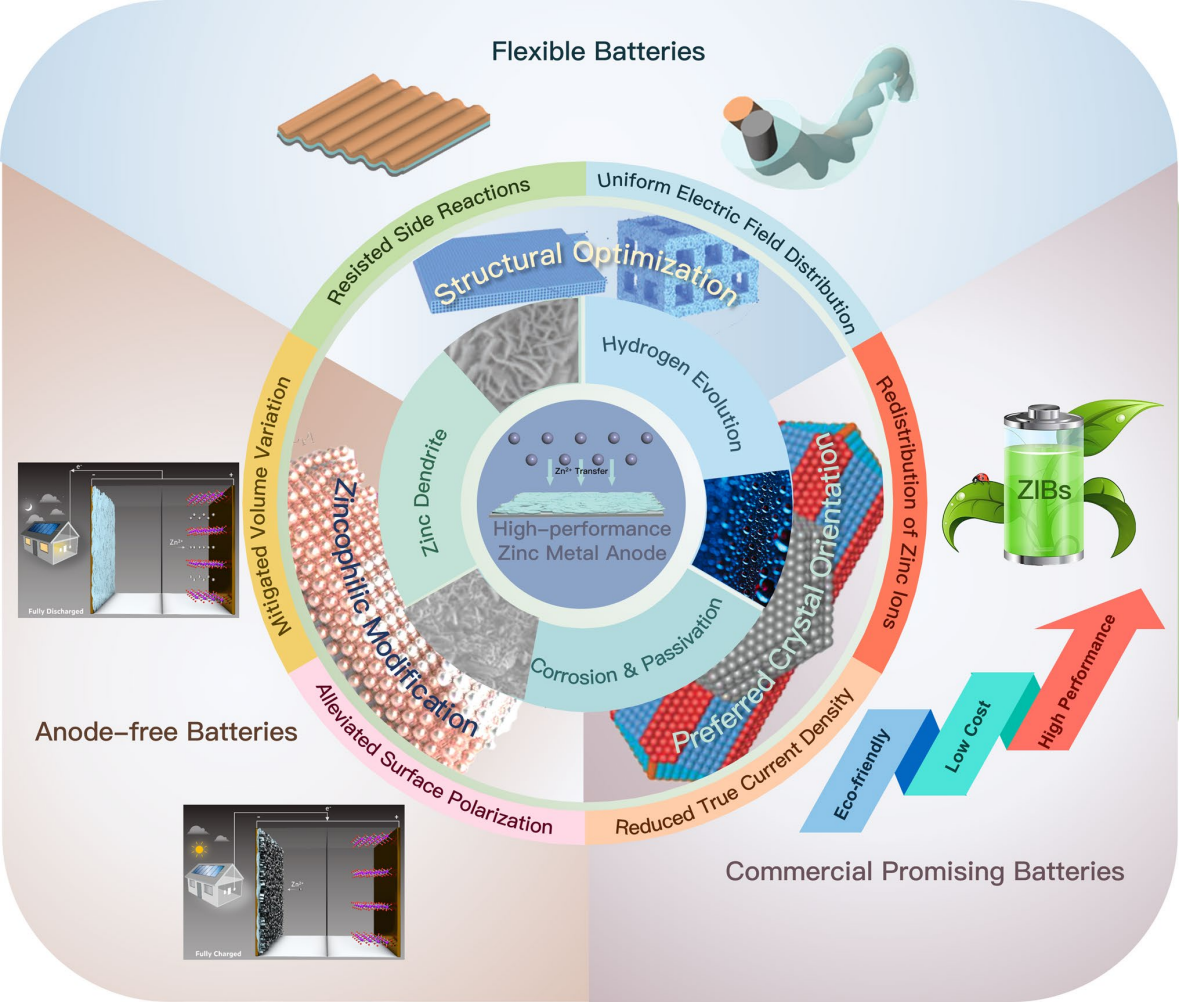
Overview of the effect of structural design and surface modification strategies on zinc anodes. Structural optimization method: Adapted from Ref. [76]. Copyright 2021, John Wiley and Sons; zincophilic modification method and corrosion and passivation: Adapted from Ref. [77, 93]. Copyright 2022, the American Association for the Advancement of Science; preferred crystal facet orientation method and zinc dendrites: Adapted from Ref. [78]. Copyright 2022, John Wiley and Sons. Anode-free battery systems: Adapted from Ref. [79]. Copyright 2021, American Chemical Society; flexible batteries: Adapted from Ref. [80]. Copyright 2020, John Wiley and Sons

In recent years, various effective strategies have been proposed to address the challenges associated with zinc anodes. These strategies can be broadly categorized into three types: electrolyte modification [17, 62, 63], zinc anode interfacial modification layer [54, 64, 65], and CC optimization [14, 66, 67]. Electrolyte modification aims to optimize the arrangement of Zn^2+^ in the solvent to reduce the impact of active water molecules on the Zn^2+^ deposition process [68–70]. Interfacial layer modification, on the other hand, aims to induce the de-solvation of Zn^2+^ to promote uniform deposition and prevent direct contact between active water molecules and zinc anodes [71, 72]. These methods have proved effective in alleviating the problems faced by zinc anodes and improving the cycle life of ZIBs. However, the increased cycle life is often achieved by adding excess zinc, whose actual depth of charge and discharge (DOD) may be only 2% to 3%, and excess zinc reduces the mass energy density of the battery [73]. By implementing structural design or surface modification on the anode CC, it is possible to enhance the utilization rate and depth of discharge of zinc anodes. This can be achieved in two ways. Firstly, adjusting the surface state of the CC can improve the current distribution on collector's surface, leading to uniform deposition of zinc. Secondly, regulating the surface HER overpotential can suppress side reactions and improve the overall performance of the battery. These approaches offer promising avenues for optimizing the practicality and extending the life span of ZIBs [74, 75] (Fig. 2).

The mechanisms of collector modification can be broadly classified into (1) zincophilic modification to induce uniform zinc morphology; (2) the optimization of the structure of the collector to achieve a better deposition effect; and (3) the use of crystal orientation preferred materials to construct the desired crystal facet orientation. Each of these three mechanisms will be described below.

### Zincophilic Modification

As discussed in Sect. 2, mesoporous zinc networks are formed as zinc clusters expand around absorbed zinc single-atom sites during the deposition process. The uniformity of this deposition process is strongly influenced by the energy of the surface, as new nuclei may form or grow on existing nuclei depending on the surface energy and distribution of nucleation sites [81]. The concept of “zincophilicity,” migrated from the “lithiophilicity” first proposed by Liu et al. [82], has emerged as a key factor in this process, referring to the binding and adsorption energy (*E*_a_) between Zn and the material surface [83]. Based on predictions via density functional theory (DFT) computation, researchers could conduct preliminary projections to the absorption energy and zincophilicity of concrete materials. This energy is in accordance with Eq. (8) [52]:

8 $$E_{{\text{a}}} = E_{{{\text{Total}}}} - E_{{{\text{Zn}}}} - E_{{{\text{CC}}}}$$

in which the ${\text{E}}_{\text{Total}}$, ${\text{E}}_{\text{Zn}},$ and ${\text{E}}_{\text{CC}}$ denote the energy of the zinc absorbed on the surface of CC, the energy of zinc atom, and the energy of CC, respectively. Materials with poor zincophilicity tend to have a higher nucleation overpotential and insufficient active sites for bulk deposition of zinc, leading to an increased zinc grain size tendency of dendrite formation [84].

To address this, zincophilicity modification of the CC can enhance the uniformity of zinc deposition and reduce dendrite formation. Additionally, adjusting the HER overpotential on the CC's surface can effectively suppress side reactions, further improving the performance of the battery. The extensively studied zincophilic modification sites can be divided into metal-based sites, which tends to form alloys with zinc, and nonmetal-based sites, which have abundant electron-rich defects that could bond with Zn^2+^.

#### Metal-Based Zincophilic Sites

When zinc is deposited on the surfaces of metals such as Au, Ag, and Cu, which have a good zincophilicity, it tends to enter the metal lattice and form eutectic alloys such as AuZn_4_, AgZn_3_, and CuZn_5_, due to the high binding energy of zinc to these metal surfaces. Recent DFT calculations and experimental findings have confirmed that the binding energy of zinc is even higher when deposited on the surfaces of these alloys than on the zinc substrate itself [85]. In addition to enhancing the substrate's zincophilicity and promoting uniform deposition/dissolution of zinc, such modified layers are also expected to possess high surface HER overpotential to minimize the impact of hydrogen.

**Fig. 3:**
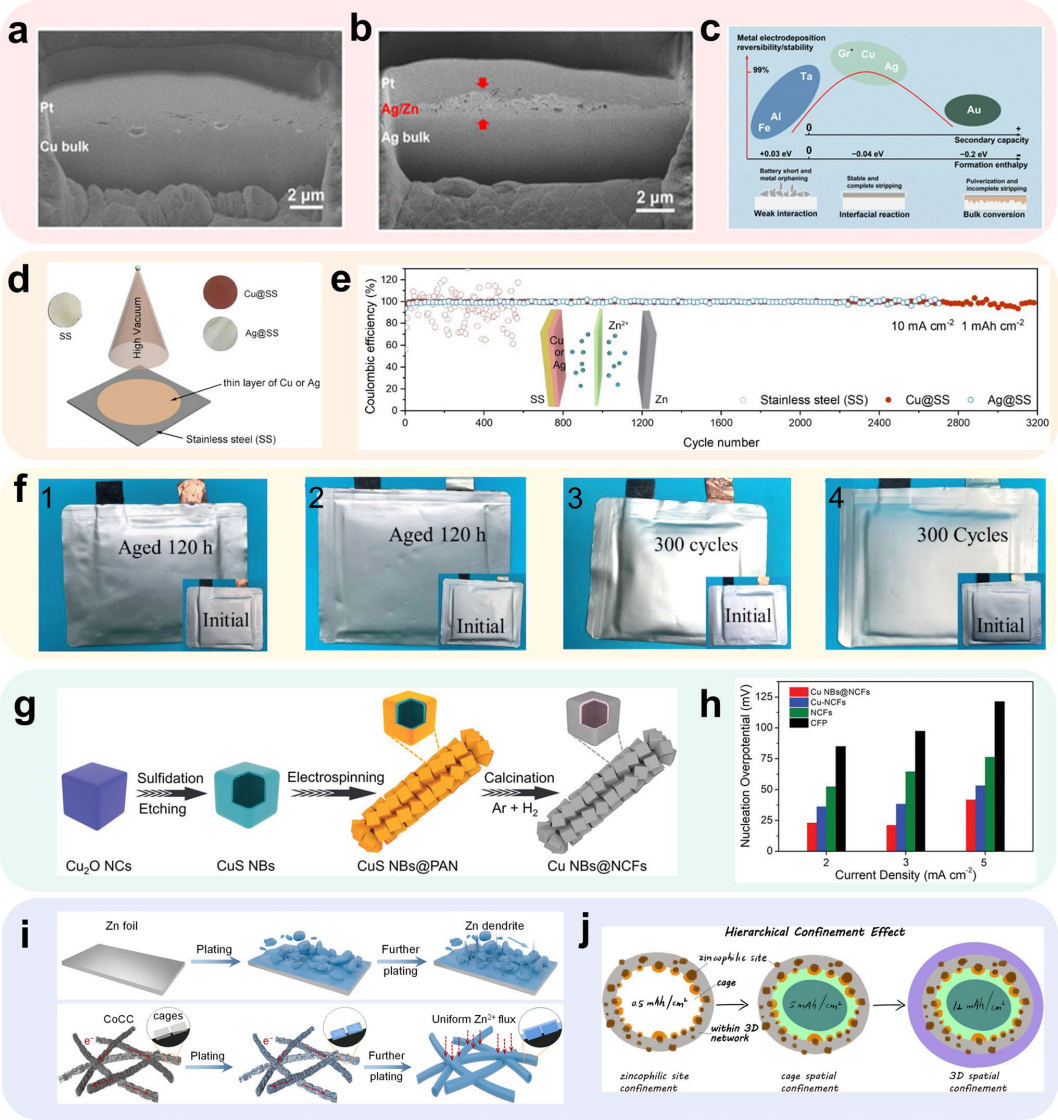
Methods using metal-based zincophilic sites. FIB-SEM characterization of zinc deposited on **a** Cu and **b** Ag; **c** qualitative volcano-shaped relation in battery anodes. Adapted from Ref. [86]. Open access; **d** schematic illustration of Ag@SS and Cu@SS; **e** Coulombic efficiencies of Zn deposition/dissolution cycles on the SS, Cu@SS, and Ag@SS substrates at 10 mA cm^− 2^ and 1 mAh cm^− 2^. Adapted from Ref. [88]. Copyright 2021, Elsevier; **f** digital images of 1 Zn-P@Cu||MnO_2_ and 2 Zn-P@Sn-Cu||MnO_2_ before and after aging and 3 Zn-P@Cu||MnO_2_ and 4 Zn-P@Sn-Cu||MnO_2_ before and after cycling. Adapted from Ref. [58]. Copyright 2021, John Wiley and Sons; **g** schematic illustration of the synthetic process of Cu NBs@NCFs; **h** nucleation overpotential of different hosts tested at current densities of 2, 3, and 5 mA cm^−2^. Adapted from Ref. [89]. Copyright 2021, John Wiley and Sons; **i** schematic illustrations of Zn deposition behaviors on zinc foil and CoCC; **j** schematic illustration on different amount of Zn deposition. Adapted from Ref. [84]. Copyright 2022, American Chemical Society

It is noteworthy when discussing the zincophilicity nature of a certain metal, there is a tendency to consider solely the deposition of Zn on its surface and ignore or downplay the influence of these metals/alloys during the dissolution process. In a study by Zheng et al. [86], the mechanisms of Zn alloying on the surface of Au, Ag, and Cu are compared when they are used as modification layers of the anode substrate. The authors have found that the alloying process of Zn on the surface of Cu resembles a pseudo-capacitive behavior, which is limited to the surface layer (Fig. 3a), while the process on Au and Ag surfaces is controlled by mass transfer and tends to generate within the substrate (Fig. 3b). Moreover, the half-cell test and in situ SEM analyses indicated that the binding energy between Zn and the Au substrate is so intense, which resulting in the formation of an AuZn_4_ interphase compound that hindered the dissolution of zinc. This incomplete dissolution of Zn may ultimately lead to the destruction of the Au substrate. Conversely, the binding energy of Zn to Cu and Ag is moderate, allowing for the vast majority of Zn entering the substrate to be reversibly dissolved without significant damage to the substrate (Fig. 3c). Other metals such as Sn [33] and In [87], which have relatively high HER overpotential on the surface and good zincophilicity, are also commonly used in metal-based modification sites.

When selecting CC substrates, cost, stability, and electrical conductivity are often the primary considerations. Metal substrates have attracted the attention of researchers due to their superior electrical conductivity, high mechanical strength, and other advantages. Various metal substrates, including copper foil [74], titanium foil [90], and alloy CCs [85, 91, 92], have been studied. Zincophilicity modification sites can be introduced onto the surface of metal substrates using various techniques, such as electroless plating [33, 93, 94] and physical vapor deposition (PVD) [85, 86, 95, 96]. For instance, Zhang et al. [88] employed vacuum thermal evaporation coater to introduce copper and silver modification layers onto the surface of steel sheets (SS), respectively, in order to achieve uniform zinc deposition (Fig. 3d). Scanning electron microscopy (SEM) images of the plated layers showed the presence of copper and silver nanoparticles on the steel sheet surface, which provided channels for the transport of Zn^2+^. Half-cell tests of Zn||Cu@SS, Zn||Ag@SS, and Zn||SS demonstrated that the nucleation overpotential of Zn^2+^ on the copper- and silver-modified steel sheets was effectively reduced (40 and 29 *vs.* 77.6 mV). The average CE of Cu@SS and Ag@SS reached 99.7% and 99.6% at 10 mA cm^−2^ and 1 mAh cm^−2^, demonstrating the excellent reversibility of zinc deposition/dissolution on the collector surface (Fig. 3e). Moreover, when using the Cu or Ag layer as a protective layer for the Zn negative electrode, X-ray diffraction (XRD) characterization revealed the formation of Zn as an alloy phase with Cu and Ag. This can significantly alter the interface between Zn and the interfacial layer, reduce the nucleation potential, and further confirm the effectiveness of using Cu and Ag as modification layers mentioned above.

Despite the high zincophilicity of copper, spontaneous electrochemical corrosion of zinc occurs on the surface of the copper collector when the battery is left aging. This phenomenon is particularly evident in zinc powder batteries, where the porous zinc powder particles are loaded on the surface of the copper foil, allowing electrolytes to penetrate at the active material/substrate interface, creating galvanic corrosion microcells mentioned in Sect. 2. These microcells caused rapid loss of active material and cell failure. In a study by Zhi et al. [58], the researchers conducted shelf-aging tests on the cell and confirmed the above idea (Fig. 3f). To address this self-corrosion problem of the zinc powder anode (Zn-P@Cu), the researchers introduced a tin modification layer on the copper foil surface using electroless plating. The corrosion microcurrent measurements and aging tests of the cell showed that the Zn-P@Sn-Cu collector exhibited more stable shelving performance and longer shelf life compared to Zn-P@Cu. Specifically, the corrosion current of Zn-P@Cu after 800 min of shelving was 10 μA cm^−2^, while the corrosion current of Zn-P@Sn-Cu was close to 0. Additionally, after 120 h of full cell aging, the Zn-P@Sn-Cu collector had only a 20% loss of active material, compared to a 50% loss of active material in Zn-P@Cu. This work has provided a feasible solution to the self-corrosion problem of the zinc powder anode and offered an optional strategy for its practical application.

Self-supporting carbon materials have been extensively studied as an alternative to metallic conducting skeletons due to their stable chemical properties, superior electrical conductivity, and porous morphology [97]. To induce uniform deposition of zinc, zincophilic metal sites can be doped into substrates in a uniformly disperse way through electrospinning [98] and PVD [85]. A CC skeleton material comprising cubic copper nanocages as zincophilic and zinc-storaging sites and nitrogen-doped carbon fibers as the conductive skeleton has been designed by Zeng et al. [89]. This material is fabricated by mixing the prepared copper nanoboxes with the electrospinning solution for spinning and subsequently carbonizing the skeleton (Fig. 3g). Similar to the three-dimensional copper skeleton, the Cu NBs@NCFs with high specific surface area have the effect of reducing the surface current density and decreasing the electrochemical polarization. The Cu NBs@NCFs have been experimentally and DFT confirmed to not only alloy with Zn, reducing the nucleation overpotential, but also promote the deposition of Zn with more (0 0 2) crystalline surfaces, optimizing the deposition morphology of Zn on the CC (Fig. 3h).

By controlling the load and distribution of metal nanoparticles in carbon fibers, the deposition position of zinc in the skeleton can be controlled for different zinc deposition amounts, improving the distribution uniformity of zinc. Metal organic frameworks (MOFs), such as ZIF-67 and MIL-88, are a series of structures formed by the coordination of organic ligand molecules and transition metal ions. A carbon skeleton with a hollow structure has been prepared by scattering ZIF-67 into the spinning solution (Fig. 3i) [84]. After pyrolysis, MOF-based zincophilic Co nanoparticles have been dispersed in the interior of the hollow skeleton. The zinc is introduced to nuclear on the Co nanoparticles during the initial nucleation process on the surface of the collector. As the deposition amount increases, the zinc gradually grows in the interior of the hollow skeleton. While a larger amount of zinc is deposited, the three-dimensional structure of the skeleton allows ion conduction and electric field dispersion to alleviate the polarization during zinc deposition (Fig. 3j). This deposition behavior with the amount of zinc deposited not only allows zinc to be uniformly dispersed in space but also provides protection for zinc from spontaneous side reactions with the electrolyte through the outer carbon skeleton.

#### Nonmetal-Based Zincophilic Sites

Carbon-based collectors are one of the extensively used substrates in anode research, owing to their unique constructions and relatively mature means of preparation, which provide good electrical conductivity and stability. Moreover, unlike metallic substrates that require casting or electrochemical deposition for their preparation, carbon-based substrates can be fabricated by various techniques, such as vapor deposition [99, 100] and organic carbonization [101], enabling variability in their morphology and physical properties, and providing a range of options for raw materials. However, unmodified carbon-based substrates are widely known for their poor zincophilicity, thereby impeding their direct application in high-performance ZIB systems [102, 103]. Hence, researchers have focused on modifying carbon materials by introducing functional sites to enhance their zincophilicity.

**Fig. 4:**
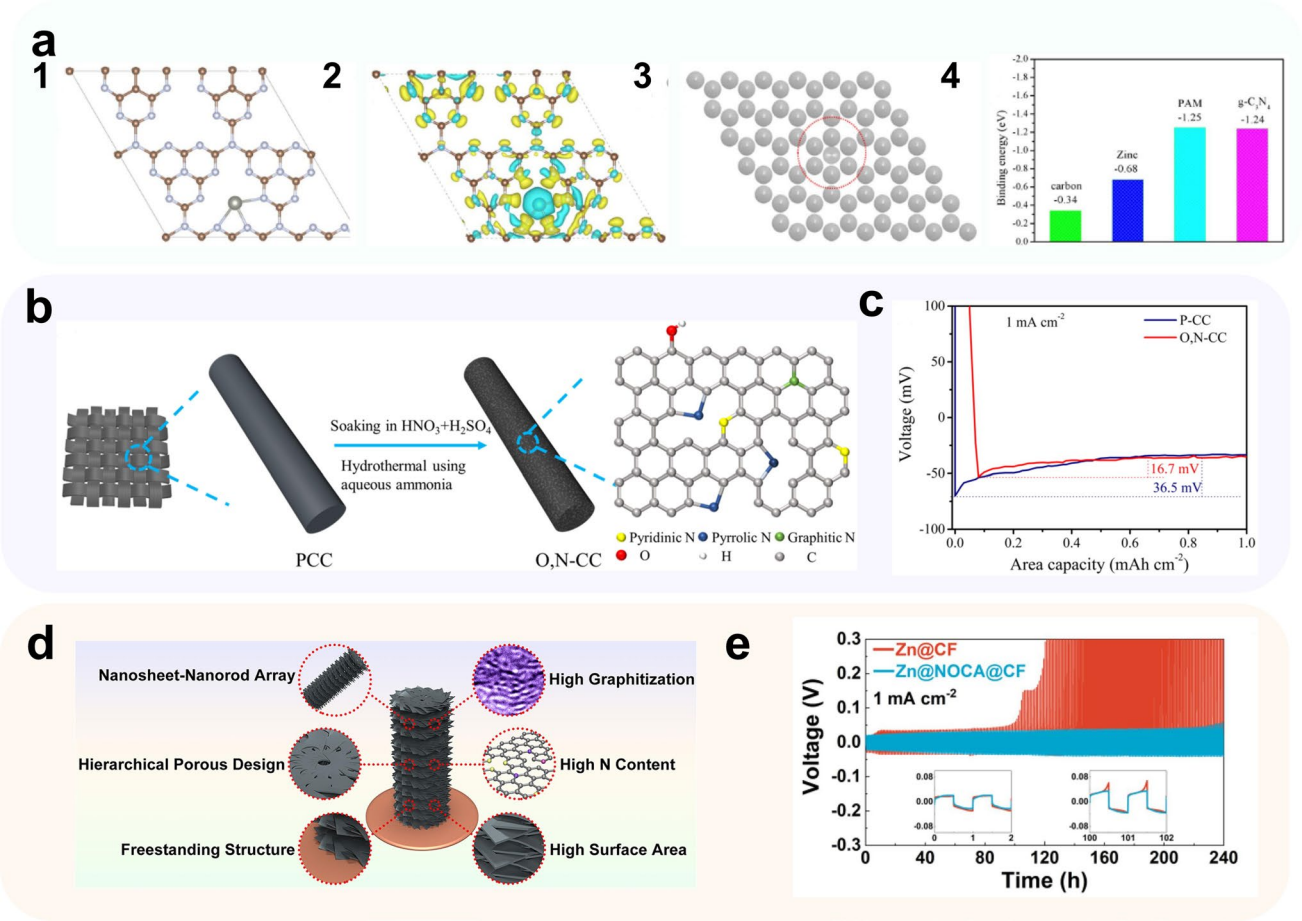
Methods using metal-based zincophilic sites. **a 1** Crystal models for calculating the binding energy of a Zn^2+^ adsorbed on g-C_3_N_4_ and **2** the corresponding charge density difference (yellow and light blue areas represent positive and negative charge differences, respectively), **3** crystal models for calculating the binding energy of a Zn^2+^ adsorbed on Zn, **4** binding energy of a Zn^2+^ with different substrates. Adapted from Ref. [105]. Copyright 2021, Elsevier; **b** schematic illustration for the preparation process of O, N-CC; **c** voltage–area capacity curve during nucleation in the case of P-CC and O, N-CC electrodes at a current density of 1 mA cm^−2^. Adapted from Ref. [103]. Copyright 2022, Elsevier; **d** schematic illustration of structural merits of NOCA@CF product; **e** voltage curves of symmetric cells with Zn@CF and Zn@NOCA@CF anodes at rates of 1 mA cm^−2^. Adapted from Ref. [106]. Copyright 2020, Elsevier

Elemental doping, such as nitrogen, oxygen, and sulfur, has been found to effectively enhance the affinity of carbon materials for Zn^2+^ [104]. Specifically, graphene-like carbon nitride (g-C_3_N_4_) with a nitrogen atomic percentage of 57% has shown a remarkable affinity to Zn^2+^ ions. Liu and colleagues [105] investigated the interaction between Zn^2+^ and g-C_3_N_4_ using DFT calculations, including binding energy and charge density difference analysis (Fig. 4a2). They modeled the interaction between Zn^2+^ and g-C_3_N_4_ (Fig. 4a1), as well as Zn foil (Fig. 4a3), using Vienna Ab-initio Simulation Package (VASP). The results showed that the binding energy of g-C_3_N_4_ with Zn^2+^ is quite large at − 1.24 eV (Fig. 4a4), indicating a strong interaction between g-C_3_N_4_ and Zn^2+^. On the contrary, the binding energy of Zn^2+^/Zn is much smaller compared to Zn^2+^/g-C_3_N_4_, at − 0.68 eV. The charge density difference model suggested that N in g-C_3_N_4_ possesses a pair of solitary p-electrons, as well as high electronegativity (*χ* = 3.04). This configuration facilitated g-C_3_N_4_ to capture Zn^2+^ in electrolytes, leading to the formation of a relatively stable intermediate an “electron donor−electron acceptor” structure. Therefore, the uniform distribution of N on the surface of g-C_3_N_4_ could induce the uniform deposition of Zn, while the abundant N on the surface provided a number of Zn nucleation sites, effectively reducing the nucleation overpotential of Zn^2+^.

Zhou et al. [103] have provided further evidence of the zincophilic property of N by introducing O and N elements onto the carbon substrate surface through the hydrothermal method (Fig. 4b). C–O functional groups can coordinate with Zn^2+^ in the electrolyte, directing its two-dimensional diffusion upon the surface. Meanwhile, N-doped sites, such as pyrrole N, pyridine N, and graphite N, can reduce the nucleation potential of Zn on the collector surface, creating more Zn nucleation sites that promote uniform Zn nucleation. This “diffusion–nucleation” synergy induced by oxygen and nitrogen atoms on the collector surface optimizes the charge transfer and deposition/dissolution kinetics of Zn^2+^ on the electrode surface. The half-cell test results reveal that the O, N-functionalized carbon cloth with Zn (O,N-CC@Zn) has a significantly smaller Zn^2+^ nucleation overpotential of 16.7 mV compared to unmodified plain carbon cloth with Zn (P-CC@Zn) and Zn foil (36.5 and 64.2 mV, respectively) (Fig. 4c).

Leveraging the diverse ligand molecules present in MOFs, researchers have discovered that heat treatment can lead to the creation of unique carbon materials with distinct morphologies and elemental dopants. By heat treating the substrate with surface loading ZIF-8, An et al. [106] achieved a dendrite-free zinc deposition process on copper foam. Through vacuum pyrolysis, a stacked nanoarray of graphite layers was produced by carbonizing ZIF-8 grown on copper foam via hydrothermal methods (Fig. 4d). The process resulted in the release of gaseous Zn and increased porosity in the nanostructure. This, in turn, resulted in a greatly increased specific surface area, leading to an effective decrease in the true current density. The rodlike nanoarrays induced a uniform flux of Zn^2+^, further enhancing the anode's performance. Moreover, the introduction of O and N dopants, comprising approximately 3% and 20% of the total atoms, respectively, increased the hydrophilicity and zincophilicity of the collector. As a result, the homogeneous mass transfer process and the improved substrate zincophilicity synergistically enabled the uniform deposition of Zn on the N/O dual-doped carbon array tightly grown on Cu foam (NOCA@CF) surface by a dendrite-free morphology. Compared to the Zn@CF electrode, the symmetric cells assembled with Zn@NOCA@CF exhibited better stability at current densities of 1 and 2 mA cm^−2^, achieving a cycle life of over 300 cycles while maintaining an average Coulomb efficiency of approximately 95.7% and 95.3%.

**Fig. 5:**
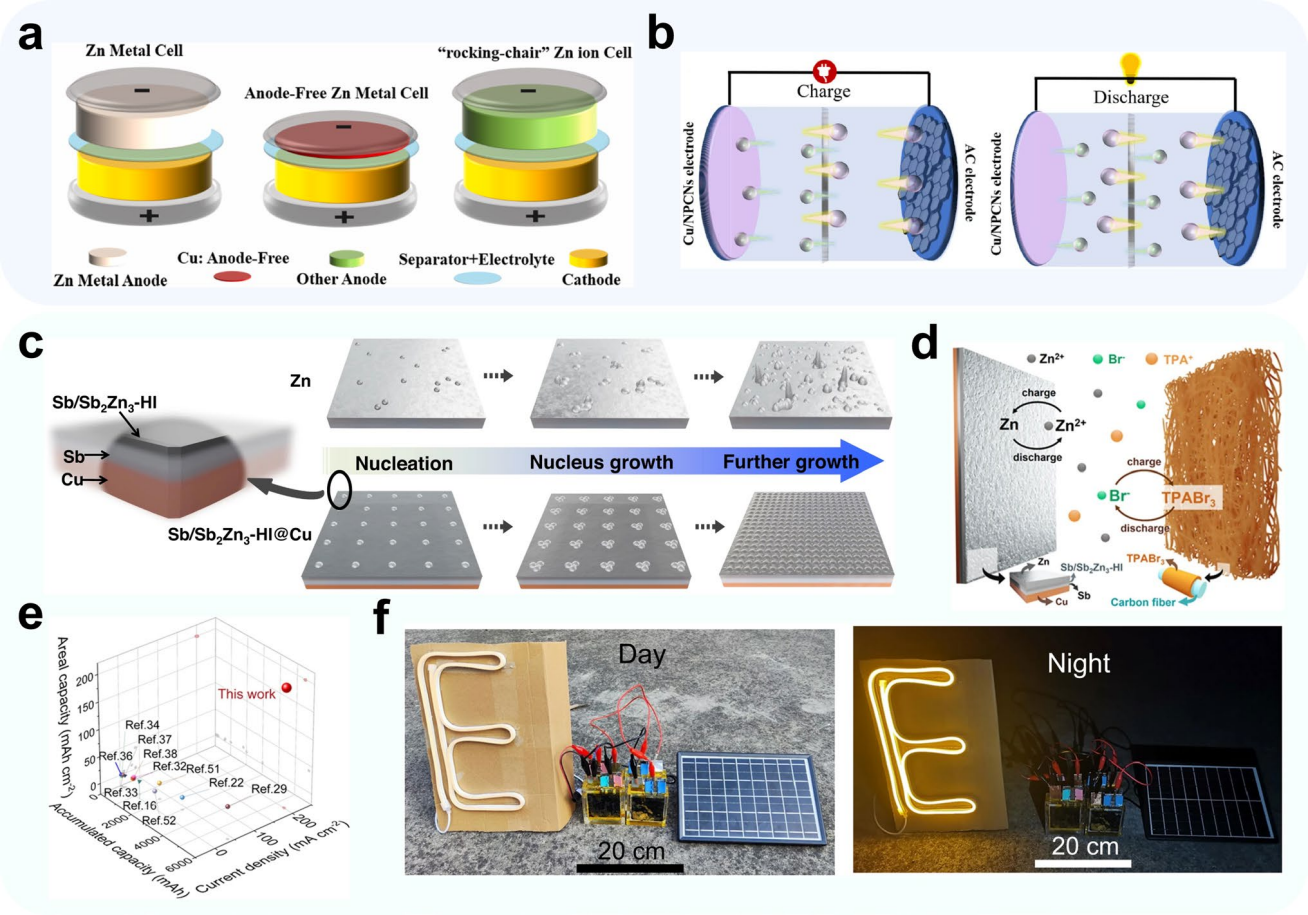
Anode-free battery systems.** a** Schematic illustration of three different battery configurations; **b** schematic demonstrations of the anode-free Zn-ion capacitor at fully charged and discharged states. Adapted from Ref. [109]. Copyright 2022, Elsevier; **c** schematic diagrams of Zn electrodeposition on Zn and Sb/Sb_2_Zn_3_-HI@Cu substrates; **d** a schematic diagram of the anode-free Zn–Br_2_ battery; **e** a summary of Zn-free electrodes for Zn deposition/dissolution in terms of areal capacity, current density, and accumulated capacity; **f** solar-powered battery energy storage system at day and night. Adapted from Ref. [110]. Open access

Simultaneously, the enhanced reversibility of the zinc deposition/dissolution mechanism on the substrate surface owing to the presence of zincophilic sites results in reduced zinc loss during battery operation. This, in turn, leads to superior deep charge/discharge performance and facilitates the construction of the intricate "anode-free" battery system [79, 107]. The "anode-free" battery system is a groundbreaking innovation that comprises solely of cathodes (cathode active materials with pre-embedded Zn, in zinc batteries, to be specific), electrolyte, and anode CC, absence of any anode active material (Fig. 5a). This unique feature facilitates an increase in the mass energy density of the battery, as it eliminates the need for high-density anode active material [108]. In a recent study, Liu et al. [109] successfully constructed zincophilic sites by integrating nitrogen-doped porous carbon nanocages onto the copper foil. They utilized this technique to assemble an anode-free zinc-ion hybrid capacitor, which displayed remarkable performance characteristics (Fig. 5b). Specifically, the hybrid capacitors demonstrated higher energy density (106 mAh g^−1^ and 101 Wh kg^−1^) and superior capacity retention (98% for 2000 cycles) when operated at a current density of 1 mA cm^−2^, thanks to the regulating influence of the zincophilic-modified layer. Chen et al. and their colleagues [110] have made a significant breakthrough by constructing an Sb/Sb_2_Zn_3_ heterostructure interface on the copper foil's surface, using the alloying of zinc with antimony (Fig. 5c, d). By capitalizing on the zincophilic and uniform electric field distribution characteristics of the Sb/Sb_2_Zn_3_ heterostructure interface during zinc deposition, the resulting zinc anode attained an area capacity density of 200 mAh cm^−2^. Furthermore, the anode-free carbon felt||Sb/Sb_2_Zn_3_-HI@Cu full cell, utilizing TPABr and ZnBr_2_ as the electrolyte, demonstrated a remarkable energy density of 274 Wh kg^−1^ (Fig. 5e). In order to investigate the practical applications of this system, the research team also constructed a prototype soft pack cell and evaluated its potential for energy storage in solar cells (Fig. 5f).

In conclusion, the introduction of zincophilic sites on the collector surface can effectively enhance the uniformity of zinc deposition, thereby delaying the fatal impact of dendrite issues on cell operating time. Moreover, the implementation of zincophilic sites can functionalize the CC surface and effectively suppress side reactions, such as HER. In terms of selecting zincophilic substrates, there are several feasible approaches, including utilizing commonly used zincophilic metals or alloys, carbon-based materials, and drawing from the experience of electrodeposition zinc. Additionally, combining theoretical calculations with experiments to screen new materials [83] is a promising avenue for exploration. The electrochemical performance in the Zn||CCs half-cells of some of the substrates mentioned above is summarized in Table S1.

### Structural Optimization

The insufficient specific surface area of conventional two-dimensional structured CCs limits the contact area between the anode active material and electrolytes; thus, the limited number of active sites causes a poorer electrochemical performance and is more prone to dendrites under a large rate of deposition/dissolution reaction [111]. Moreover, the uneven distribution of current density on the collector's surface causes certain regions to experience densification during zinc deposition, exacerbating the severity of zinc dendrite issues on 2D surfaces [112].

In recent years, there has been growing interest in the design of three-dimensional CCs [113, 114]. These collectors offer a high specific surface area, which leads to a lower local current density on the surface. This, in turn, prolongs the Sand's time (*τ*) for dendrite formation and delays the generation of zinc dendrites [115, 116]. Moreover, the increase in the specific surface area provides more zinc deposition sites and a larger electrode/electrolyte contact area, facilitating high-rate cycling and deep charge/discharge performance [117–119]. The porous structure of these collectors also provides ample space for large amounts of zinc storage, reducing volume fluctuation during operation and increasing its safety [120].

In terms of their structure, three-dimensional (3D) CCs can be classified into three types: commercial porous CCs, 3D zinc alloy, 3D printed structures, and gradient-designed materials.

#### Commercial Porous CCs

In recent years, commercially available porous substrates, including metal mesh, foam metal, and carbon fiber materials, have been extensively utilized in battery CCs, catalyst carriers, and other fields.

**Fig. 6:**
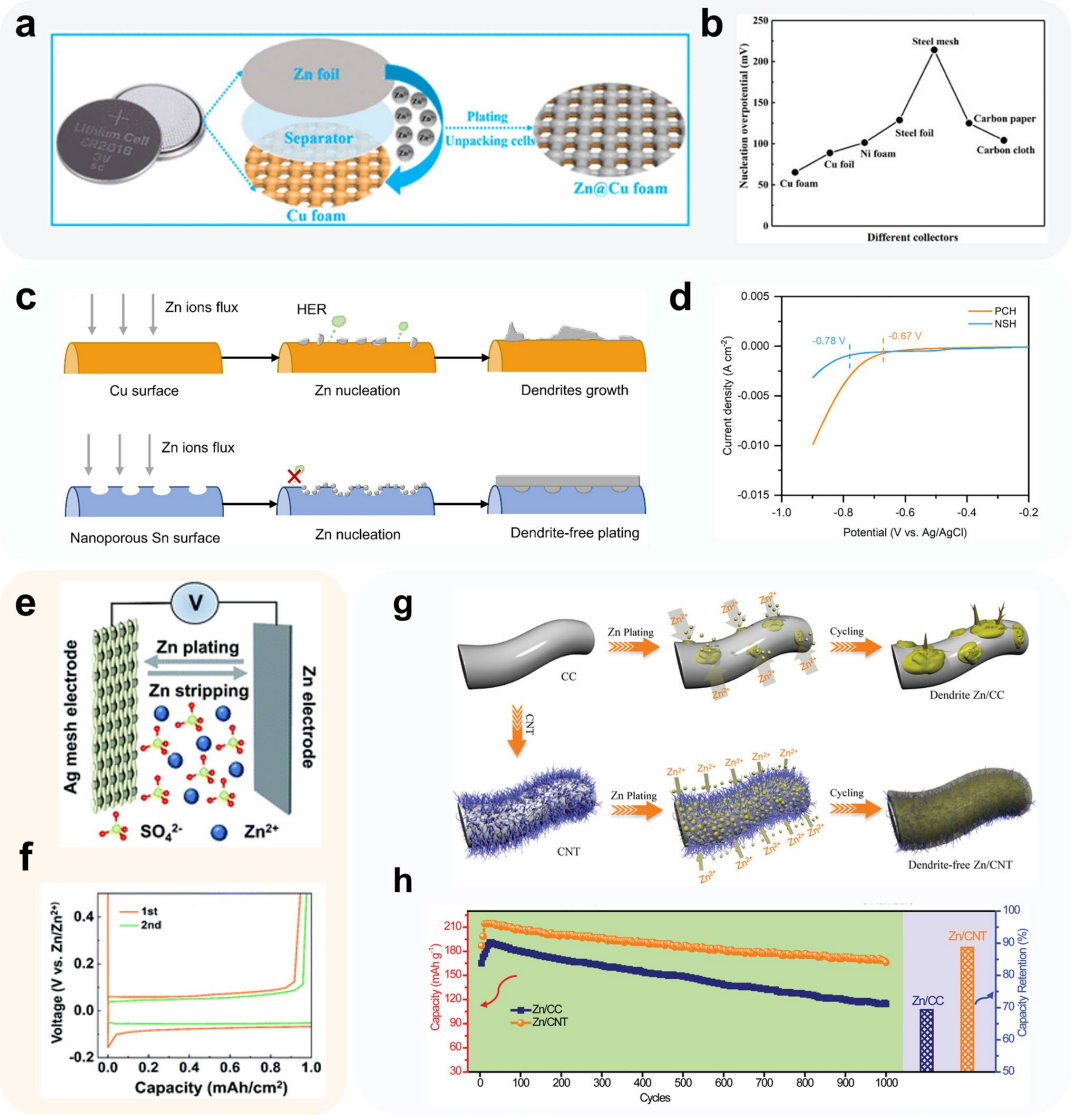
Methods using commercial porous materials.** a** Schematic illustration of the preparation process of the Zn@Cu foam anode; **b** Zn nucleation overpotentials of different collectors. Adapted from Ref. [121]. Copyright 2019, American Chemical Society; **c** schematic of Zn nucleation and deposition on PCH and NSH; **d** LSV curves of PCH and NSH in 0.1 M H_2_SO_4_ at 1 mV s^−1^. Adapted from Ref. [122]. Copyright 2021, Elsevier; **e** schematic illustration of the cell configuration used for evaluating the cyclic performance of Zn metal; **f** first two cycles of Zn deposition/dissolution curves on Ag mesh at 5 mA cm^−2^. Adapted from Ref. [123]. Copyright 2022, Royal Society of Chemistry; **g** schematic illustrations of Zn deposition on CC and CNT electrodes; **h** cycling performance collected at 20 mA cm^−2^ of the Zn||MnO_2_ batteries with Zn/CC and Zn/CNT anodes. Adapted from Ref. [124]. Copyright 2019, John Wiley and Sons

Copper-based 3D collectors, such as copper mesh and copper foam, have gained popularity as effective and affordable materials for zinc anodes due to copper's favorable zincophilic properties and relatively low cost. In a study by Shi et al. [121], the surface Zn^2+^ nucleation overpotential of various 3D materials, including carbon cloth, carbon paper, metal mesh, and foam metal, was compared when used directly as CCs for ZIBs anodes (Fig. 6a). The researchers found that copper foam exhibited the lowest surface zinc nucleation overpotential, measuring approximately 65.2 mV at 3 mA cm^−2^, among the commonly used CCs (Fig. 6b). Moreover, copper foam CCs have demonstrated superior stability and performance in full cells.

Although commercially available 3D CCs, such as copper foam, copper mesh, or nickel foam, offer a higher specific surface area, the number of Zn^2+^ nucleation sites on their surfaces remains inadequate for high-rate charge/discharge cycles. Consequently, a current research trend involves the introduction of zincophilic sites on the substrate surface to further functionalize the collector. While the surface of copper foam exhibits good zincophilicity, the hydrogen evolution activity on unmodified Cu-based collectors remains relatively high. This phenomenon is evident in Zn||Cu half-cells, where it results in reduced CE and a shortened cell shelf-aging life [125]. To address this issue, Zhao et al. [122] have employed a ligand-assisted replacement reaction to modify a porous tin layer on the surface of copper mesh (Pristine Cu mesh Host, PCH) to enhance cycle life and inhibit HER and self-corrosion of Zn (Fig. 6c). The resulting nanoporous Sn host (NSH) provided more uniform nucleation sites for Zn deposition, avoiding random nucleation on the Cu mesh surface. The deposition overpotential of Zn on the NSH substrate (22 mV under 0.5 mA cm^−2^) is significantly smaller than that on the PCH surface (40 mV under 0.5 mA cm^−2^). Furthermore, despite the increased specific surface area of the copper network loaded with the tin-modified layer compared to the unmodified copper network, the current density of surface HER is still much lower than that of PCH in the linear sweep voltammetry (LSV) test, thanks to the high HER overpotential of the tin layer (Fig. 6d). The deposition morphology of zinc on both substrate surfaces, as observed by SEM, further confirmed these findings.

Xue et al. [123] conducted research on the direct use of zincophilic silver networks as anode CCs (Fig. 6e). As discussed in Sect. 3, the formation of Ag-Zn alloy was found to effectively reduce the difficulty of Zn^2+^ nucleation and promote Zn deposition. In their study, they confirmed that this alloying process has a positive impact on both the optimization of Zn deposition morphology and the suppression of side reactions. In their experiments on Ag mesh||Zn half-cell, no significant Zn^2+^ nucleation overpotential was observed for zinc deposition under a 5 mA cm^−2^/1 mAh cm^−2^ cycle condition (Fig. 6f). Additionally, the anode exhibited excellent compatibility and stable cycling performance in a dual-ion battery test matched with LiFePO_4_ cathode.

Zeng et al. [124] utilized electrodeposition to grow carbon nanotubes in situ on the surface of carbon cloth, which possesses excellent flexibility and chemical stability (Fig. 6g). By comparing the zinc deposition behavior on the surface of pristine carbon cloth (CC) and carbon cloth with carbon nanotubes grown in situ on the surface (CNT), combined with Maxwell electric field distribution simulations, they demonstrated that the uniformly distributed carbon nanotube arrays on carbon cloth could, on the one hand, increase the specific surface area of carbon cloth, reduce the electric field intensity on the surface and optimize the electric field distribution, and on the other hand, optimize the diffusion behavior of Zn^2+^ and induce the uniform distribution of Zn^2+^ on the collector surface at the initial nucleation stage. In this work, the researchers assembled a Zn/CNT symmetric cell, which stably cycled for 200 h at a current density/cycle capacity of 2 mA cm^−2^/2 mAh cm^−2^ while maintaining a low nucleation overpotential of 27 mV. Additionally, the Zn/CNT||MnO_2_/CNT full cell maintained an average CE of 97.9% and capacity retention of 88% (Fig. 6h). These results suggest that the *in situ* growth of carbon nanotubes on carbon cloth can effectively improve the performance of Zn-based batteries by optimizing the Zn deposition behavior and enhancing the stability and efficiency of the battery.

In summary, the utilization of commercial 3D structured materials as zinc anode CCs have garnered significant attention due to their numerous benefits. However, it should be noted that the uneven pore size distribution and uncontrollable surface structure of commercial 3D CCs may result in a more uneven mass transfer process. This can cause Zn^2+^ to not be uniformly distributed in the electrolyte, leading to most of the zinc being deposited on the side of the collector near the separators, which is commonly referred to as “top growth” [126, 127], despite the closer contact between the commercial 3D CC and the electrolyte. This deposition behavior reduces the effective volume of the 3D structures and increases the risk of dendrite generation, which runs counter to the original intention of introducing 3D structures [128]. Therefore, a more detailed and refined design of the structure and function of CCs is necessary to address the aforementioned issues.

#### 3D Zinc Alloy

Zinc metal can form binary or ternary alloys when combined with other metals. These alloys may give rise to collector skeletons with distinctive structures through processes such as metal etching [129] and dealloying [121]. This section will delve into the discussion of CCs that possess unique structures derived from zinc-based alloys, highlighting their individual characteristics.

**Fig. 7:**
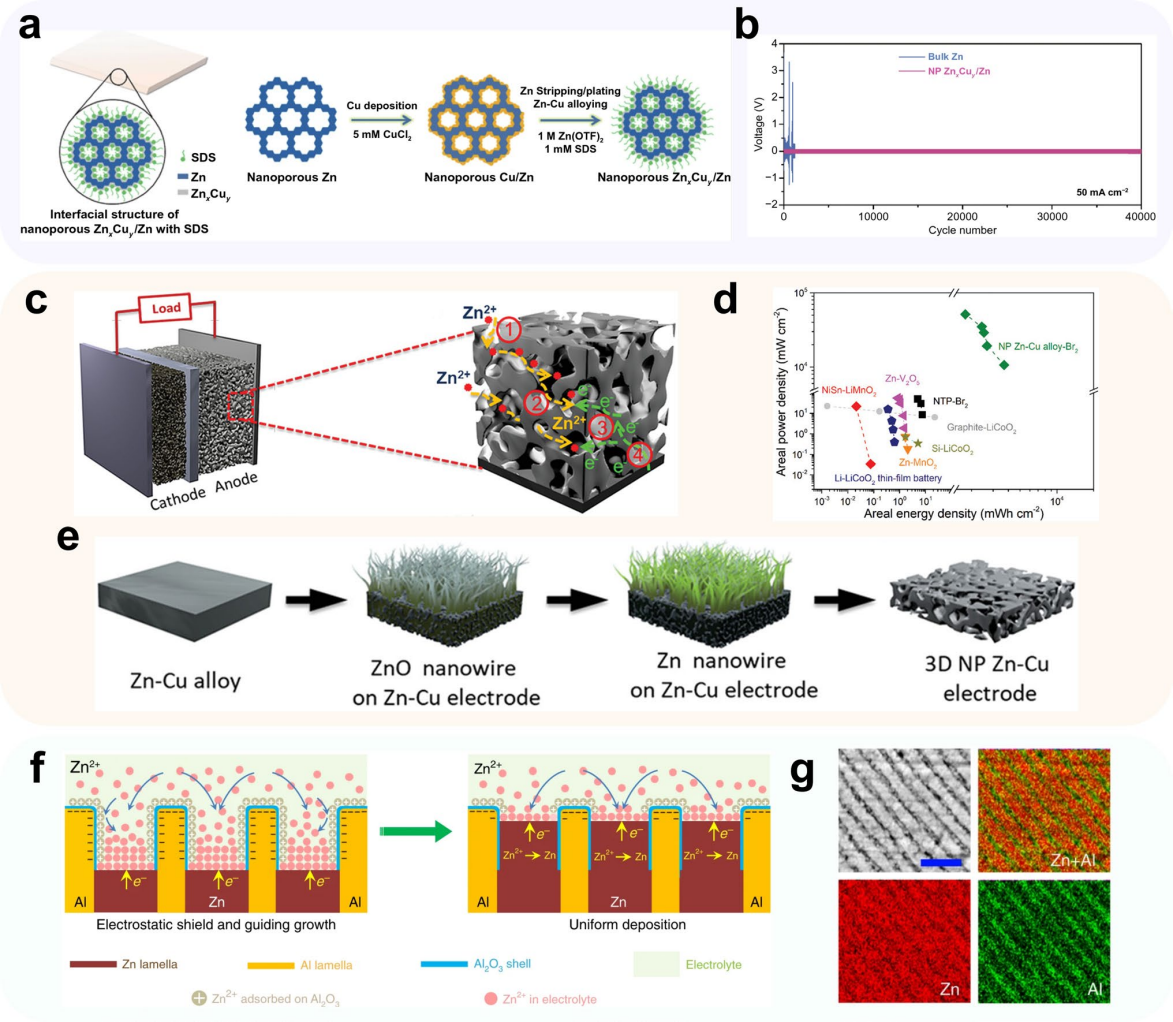
Methods using 3D zinc alloy.** a** Schematic illustration for nanoporous shell/core Zn_*x*_Cu_*y*_/Zn sheets that are fabricated by surface alloying of Cu and Zn of Cu-decorated nanoporous Zn during sodium dodecyl sulfate (SDS)-assisted electrochemical Zn deposition/dissolution cycling; **b** long-term Zn deposition/dissolution stability of symmetric cells based on nanoporous Zn_*x*_Cu_*y*_/Zn and bulk Zn electrodes in 1 mol L^−1^ Zn(OTF)_2_ with/without 1 mmol L^−1^ SDS at 50 mA cm^‒2^, respectively; Adapted from Ref. [131]. Open access; **c** schematic illustration for Zn–Br_2_ battery consisting of 3D NP Zn–Cu alloy electrode and bromine cathodes with VC/carbon textiles CC; **d** Ragone plot of area power density versus areal energy density for the RZIBs based on 3D NP Zn–Cu alloy and Br_2_ electrodes, comparing with other energy storage systems based on representative electrode materials; **e** 3D NP Zn–Cu alloy electrode fabrication process; Adapted from Ref. [130]. Copyright 2020, John Wiley and Sons; **f** schematic illustration of eutectic strategy for dendrite and crack suppression; **g** typical SEM image lamella-nanostructured eutectic Zn_88_Al_12_ alloys with lamella spacing of ∼ 450 nm and the corresponding EDS element mapping of Zn and Al. Adapted from Ref. [132]. Open access

The construction of Cu–Zn alloy CCs is a widely adopted approach to achieve uniform zinc deposition, owing to the favorable properties of Cu and Cu–Zn alloys such as good zincophilicity and high conductivity. Various methods can be employed to three-dimensionalize Cu–Zn alloys. For instance, Meng et al. [122] utilized a dealloying technique to obtain self-supporting porous structures of Zn_*x*_Cu_*y*_/Zn alloys. They achieved this by subjecting cast Zn_50_Al_50_ alloy to KOH etching, followed by in situ substitution reactions (Fig. 7a). The resulting nanoporous structure of the alloy anode facilitated a more uniform distribution of surface Zn^2+^ concentration. The introduction of surfactant SBS in the electrolyte exhibited a synergistic effect, promoting low overpotential for nucleation and highly reversible stripping of zinc (with overpotential reduced to 0 mV after 10 cycles at 0.5 mA cm^−2^). The symmetric cell based on this alloy demonstrated a cycle life of approximately 1900 h at 0.5 mA cm^−2^ and 0.5 mAh cm^−2^, while maintaining a low polarization and stable voltage profile at a current density of 50 mA cm^−2^ (Fig. 7b). In an alternative approach proposed by Liu et al. [130], a 3D nanoporous (3D NP) Zn–Cu alloy is fabricated by an electrochemical-assisted annealing thermal method (Fig. 7e). The interconnected nanopores within this alloy provided ample channels for efficient transport of Zn^2+^, while the large specific surface area offered abundant nucleation sites (Fig. 7c). As a result, the 3D NP Zn-Cu alloy electrode demonstrated excellent cycling stability. The symmetric cell based on this alloy exhibited stability for over 300 h under charge and discharge conditions of 2 mA cm^−2^ and 1.58 mAh cm^−2^, respectively. Furthermore, the Zn||Br_2_ full cell demonstrated a high energy density of 3.70 Wh cm^−2^, comparable to that of commercial lithium-ion batteries (Fig. 7d).

The ZnAl alloy is a eutectic alloy, characterized by a distinctive internal laminar structure composed of alternating Zn-rich and Al-rich phases (Fig. 4g). Wang et al. [124] conducted a creative study where they employed Zn_88_Al_12_ eutectic alloy as anode. In this design, the aluminum layer gradually exposes and undergoes surficial oxidation, forming an insulating layer of Al_2_O_3_ during the process of zinc dissolution (Fig. 7f). The regular arrangement of Al_2_O_3_/Al facilitates zinc deposition on the surface of the original zinc layer, promoting a more uniform deposition morphology and reducing the risk of zinc dendrite formation. The assembled symmetric cell based on Zn_88_Al_12_ alloy demonstrated a remarkable cycle life of over 2000 h at a current density of 0.5 mA cm^−2^. Furthermore, the Zn_88_Al_12_||K_x_MnO_2_ full cell exhibited a high energy density of 142 Wh kg^−1^. Building upon the aforementioned research, Qi et al. [125] introduced a copper mesh as a conducting skeleton into the ZnAl alloy. The resulting ZnAl@Cu mesh||V_2_O_5_ full cell achieved an impressive capacity retention of 95% after 2000 cycles at 2 A g^−1^.

#### 3D printing Materials

The utilization of 3D printing as a high-precision additive manufacturing method for energy storage devices has been extensively researched [133, 134]. Unlike commercial 3D CCs, this method allows the fabrication of 3D materials with optimized geometry that is homogeneous on a small scale. The feasibility of this process has been demonstrated in the production of lithium and sodium batteries [135]. Despite the widely recognized safety and environmental benefits of the ZIBs system, there remains a dearth of research on the 3D printed fabrication of ZIBs, which has promising applications in the field of flexible wearable devices.

**Fig. 8:**
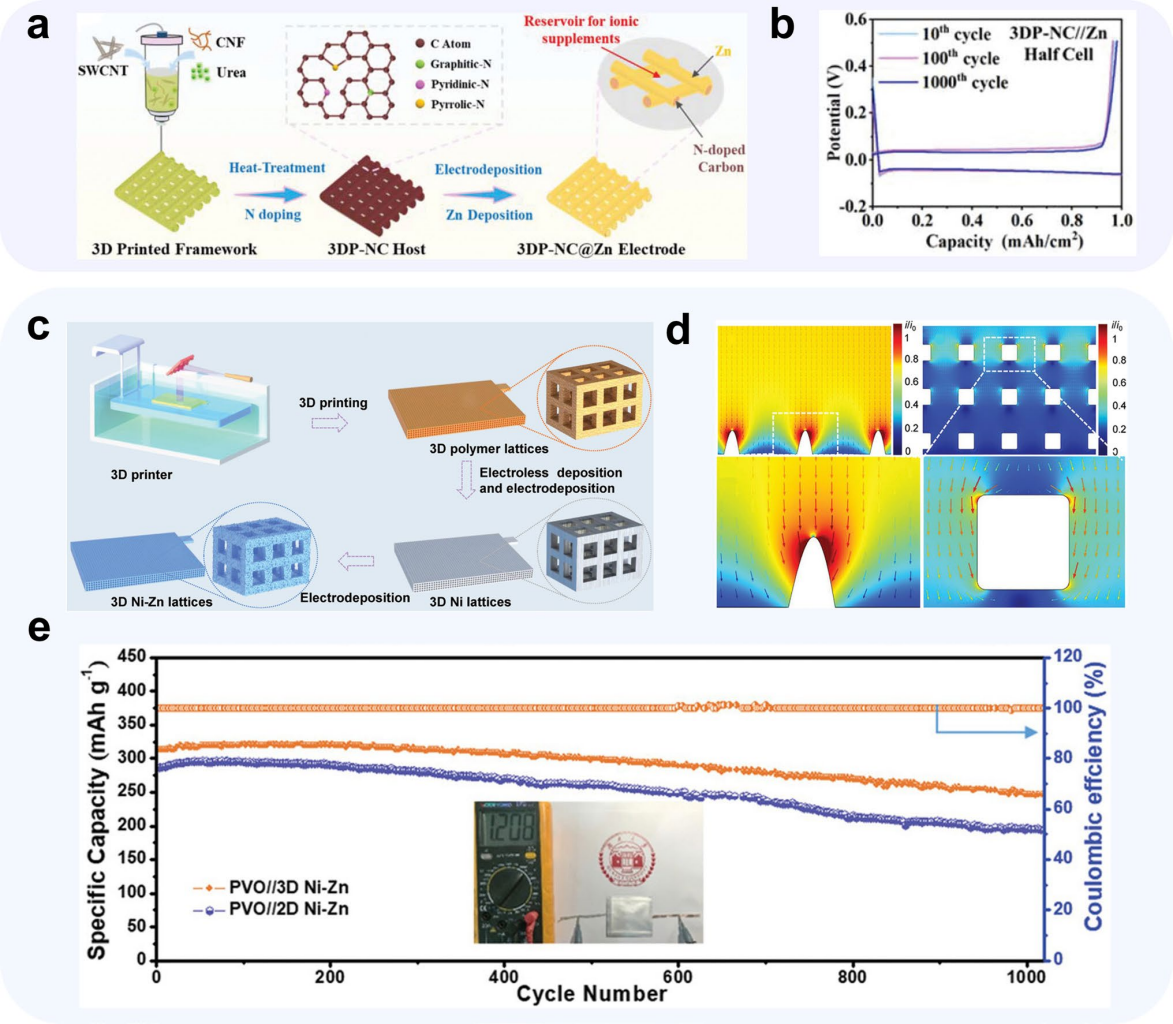
Methods using 3D print Materials.** a** Schematic illustrations of the preparation of the 3DP-NC@Zn anode; **b** voltage profiles of the 3DP-NC//Zn half-cell at 10 mA cm^−2^ at different cycles [136]. Copyright 2022, John Wiley and Sons; **c** schematic illustration of the procedure for fabricating the 3D Ni–Zn lattices. **d** Simulations of the relative intensity distributions of localized electric field for 2D Ni–Zn electrode and 3D Ni–Zn electrode; **e** cycling performance of PVO||3D Ni–Zn and PVO||2D Ni–Zn pouch cells at 10 A g^–1^. Adapted from Ref. [76]. Copyright 2021, John Wiley and Sons

Achieving targeted optimization of the three-dimensional structure of CCs is a key challenge in 3D printing technology. By controlling the structure of the collector, the mass transfer and electric field distribution can be optimized, enhancing the ability to achieve uniform distribution of Zn ions. To this end, Zeng et al. [136] designed a simple single-layer 3D printed structure and printed it using a carbon nanotube ink mixture containing nitrogen sources. After carbonization, an N-doped carbon nanotube CC (3DP-NC) was produced, which featured regular micron-sized square pores that enabled sufficient contact with the electrolyte and excellent ion diffusion ability (Fig. 8a). Moreover, these square pores served as reservoirs for the electrolyte, allowing for a large ion flux within each pore. N acted as a zincophilic site, facilitating the horizontal epitaxial growth of Zn on the surface and enabling the collector to maintain the mass transfer process in a sub-stable state, even under high current densities. The researchers employed in situ optical microscopy and multi-physics field simulations to confirm the characteristics of the CC that enable uniform distribution of the electric and ion diffusion fields. Symmetric cells with the 3DP-NC@Zn configuration exhibited a cycle life of 380 h and an ultra-low-voltage hysteresis of 7.4 mV. Asymmetric cells 3DP-NC||Zn configuration exhibited stable cycle stability and nearly 100% average Coulomb efficiency at a current density of 1 mA cm^−2^, with a zinc deposition overpotential of only 5.6 mV and stable cycling for over 1000 cycles (Fig. 8b).

Zhang et al. [76] demonstrated the effectiveness of a homogeneous cubic cage framework as a CC structure. They utilized projection microstereolithography 3D printing to print polymer molds and deposited Ni–P alloy on the mold surface by electroless plating, which served as the CC for depositing zinc on the surface and producing 3D Ni-Zn anodes (Fig. 8c). Theoretical simulations conducted using COMSOL software confirmed that the surface current density on the 3D pore structure was lower than the current density inside the pore (Fig. 8d). This current density distribution enabled the 3D Ni–Zn symmetric cell to maintain a stable cycle for over 350 h under 2 mA cm^−2^/5 mAh cm^−2^. Moreover, tests of the PVO||3D Ni–Zn full cell showed that an 80% capacity retention (314 mAh g^−1^) after 1000 cycles, even at a current density of 10 A g^−1^ (Fig. 8e).

#### Gradient-Designed Materials

Gradient CCs are structures that possess a gradient of specific properties in the horizontal or vertical direction of the CC, such as affinity gradient, conductivity gradient, and grain size gradient. The primary purpose of using a gradient collector is to deposit zinc first at the bottom of the collector, away from the separator, and gradually grow “upwards” as the amount of zinc accumulates. This approach has two key benefits: it maximizes the utilization of the space inside the three-dimensional CCs, thereby increasing the area-specific capacity of the anode, and it reduces the risk of separator penetration by dendrites that may form on the surface during the prolonged battery operation [137]. Of the gradient CCs discussed above, conductivity and affinity gradients can be achieved more easily by applying functional coatings to one side of the collector, either separately or simultaneously [138, 139].

**Fig. 9:**
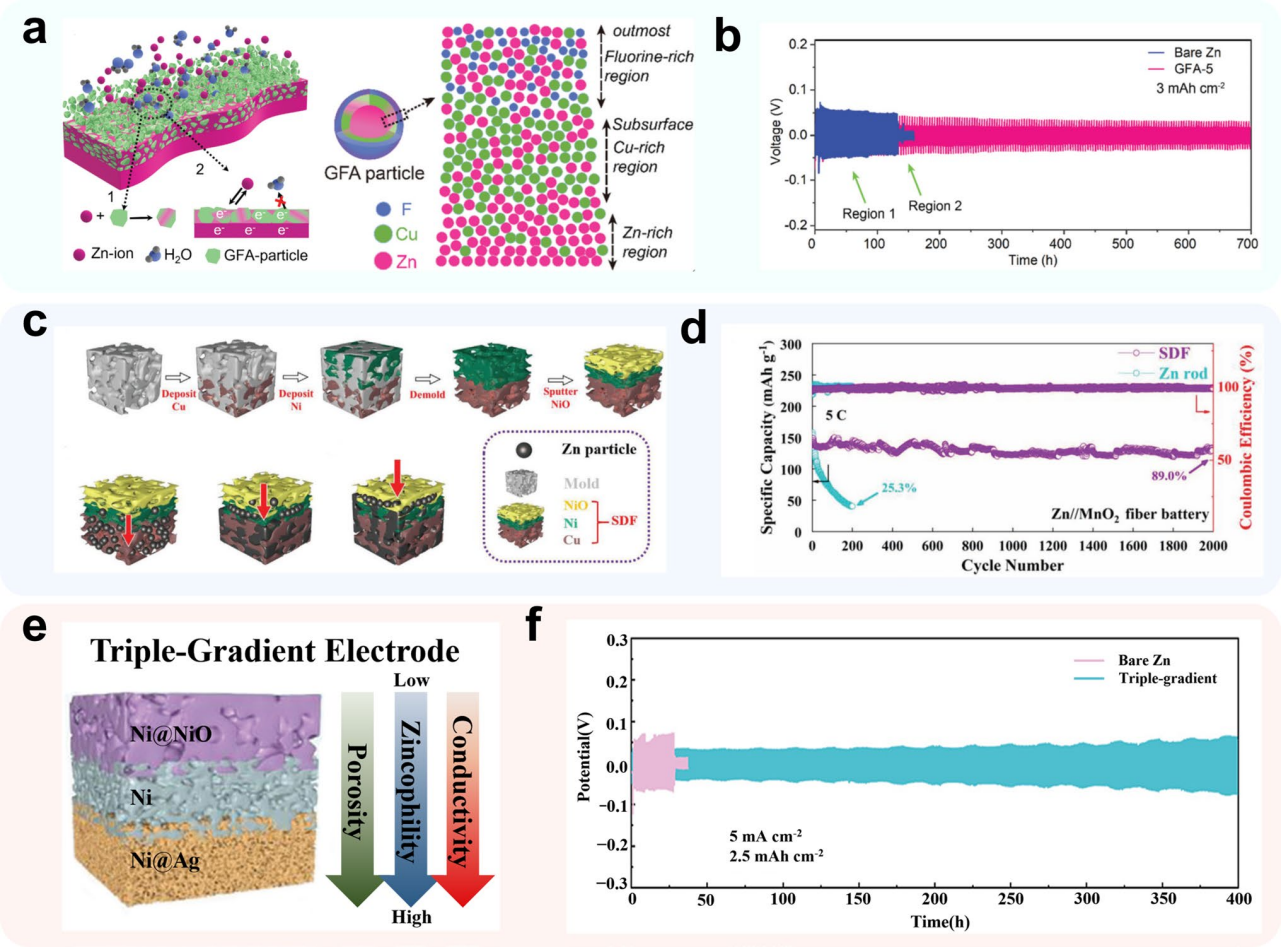
Methods using gradient-designed materials.** a** Schematic of Zn-ion transfer and the electron flow pathway during the Zn deposition process on GFA-5; **b** voltage profiles of GFA-5 cycling with a capacity of 3 mAh cm^−2^ at 3 mA cm^−2^. Adapted from Ref. [142]. Copyright 2019, Royal Society of Chemistry; **c** prepared procedures of innovative tri-layer frame collector (SDF) structure. Oriented deposition of Zn particles on SDF from Cu layer to Ni layer and eventually to NiO layer; **d** cycling performance comparison of the fibrous Zn//MnO_2_ batteries employing SDF and Zn rod anodes at 5 C. Adapted from Ref. [143]. Copyright 2021, John Wiley and Sons; **e** schematic diagram for the triple-gradient electrode; **f** cycling performance of bare Zn and triple-gradient Zn anode symmetric cells. Adapted from Ref. [144]. Copyright 2022, John Wiley and Sons

To achieve conductivity gradients, metal oxides such as Al_2_O_3_ [140] or insulating polymer coatings such as poly(vinylidene difluoride-hexafluoropropylene) (PVDF-HFP) [140, 141] are commonly used in lithium metal anode CCs to provide electronic insulation on the upper surface. However, insulative polymer coatings usually come with hydrophobic properties, which pose challenges for aqueous electrolytes to form good interfacial contacts with the CC, and impede the mass transfer process. In aqueous zinc-ion batteries, Liang et al. [142] have developed a conductivity gradient zinc anode CC using a fluoride alloy phase (GFA) with an internal spatial gradient as a coating layer. As depicted in Fig. 9a, when zinc is deposited on the collector surface, it first undergoes a replacement reaction with CuF_2_ on the GFA surface to produce ZnF_2_ with insulating properties. The replaced copper particles act as zincophilic sites and conductive skeleton, while inside the coating layer, the excess deposited Zn forms Cu–Zn alloy with Cu, inducing uniform deposition and lateral growth of zinc to prevent dendrite generation. Furthermore, the insulating property of ZnF_2_ also suppresses the HER of active water molecules from the anode surface, thus imparting better stability to the Zn anode. This innovative design enables stable cycling of the Zn anode for 700 h at a current density and capacity density of 3 mA cm^−2^/3 mAh cm^−2^ (Fig. 9b).

To achieve a zincophilic gradient, a combination of decreasing zincophilicity on the upper surface and increasing it on the lower surface is often utilized. In one example, Shen et al. [143] used NiO@Ni Foam as a zincophobic upper surface, Ni Foam as an intermediate layer, and zincophilic Cu Foam as a lower surface to construct a collector with a zincophilic gradient (Fig. 9c). The NiO surface not only acts as a modification layer to reduce zincophilicity, but also has hydrophilic and dielectric properties that can enhance interfacial contact between the electrode and electrolyte and reduce the occurrence of side reactions that produce hydrogen. As depicted in Fig. 2c, Zn preferentially deposits on the lower Cu surface and gradually grows upwards as the deposition volume increases. The gradient-based fibrous Zn-ion battery exhibited a capacity retention of 89.0% after 2000 cycles at a rate of 5C (1C = 308 mA g^−1^), which is an order of magnitude higher than that of the pristine Zn-fiber-based fibrous Zn-ion battery (Fig. 9d).

Guan et al. [144] have developed a unique collector that integrates three gradients, namely porosity, conductivity, and zincophilicity, to optimize the zinc-ion deposition process on the surface of the CC. The CC is based on a nickel foam substrate, and includes layers of NiO@NF, NF, and Ag@NF, with varying pore sizes, pressed together using the roller press method to create a piece of electrode. The collector structure, as shown in Fig. 9e, facilitates the optimization of the electric field distribution, ion transport, and deposition sites of Zn^2+^ on the surface of the collector. The NiO@NF layer, which is the topmost layer, has dielectric properties and the lowest electric field intensity on the surface, the middle layer of Ni has conductivity, while the Ag@NF layer at the bottom has better conductivity and the highest electric field intensity. The CC's zincophilic gradient varies with each layer. The uppermost layer of NiO has low zinc binding energy, the middle layer of Ni has moderate affinity energy for zinc, and the Ag layer has high zinc affinity energy that induces uniform zinc deposition. The collector's ion transport gradient is achieved by using different pore sizes in each layer. The NiO@NF layer has the largest pore size on the surface, which favors the longitudinal diffusion of Zn^2+^, while the Ag@NF layer at the bottom has the smallest pore size which provides abundant nucleation sites for zinc deposition. This triple-gradient zinc anode shows remarkable stability, operating continuously for over 400 h under a high current density/capacity of 5 mA cm^−2^/2.5 mAh cm^−2^ (Fig. 9f) and for more than 250 h under 10 mA cm^−2^/1 mAh cm^−2^.

**Fig. 10:**
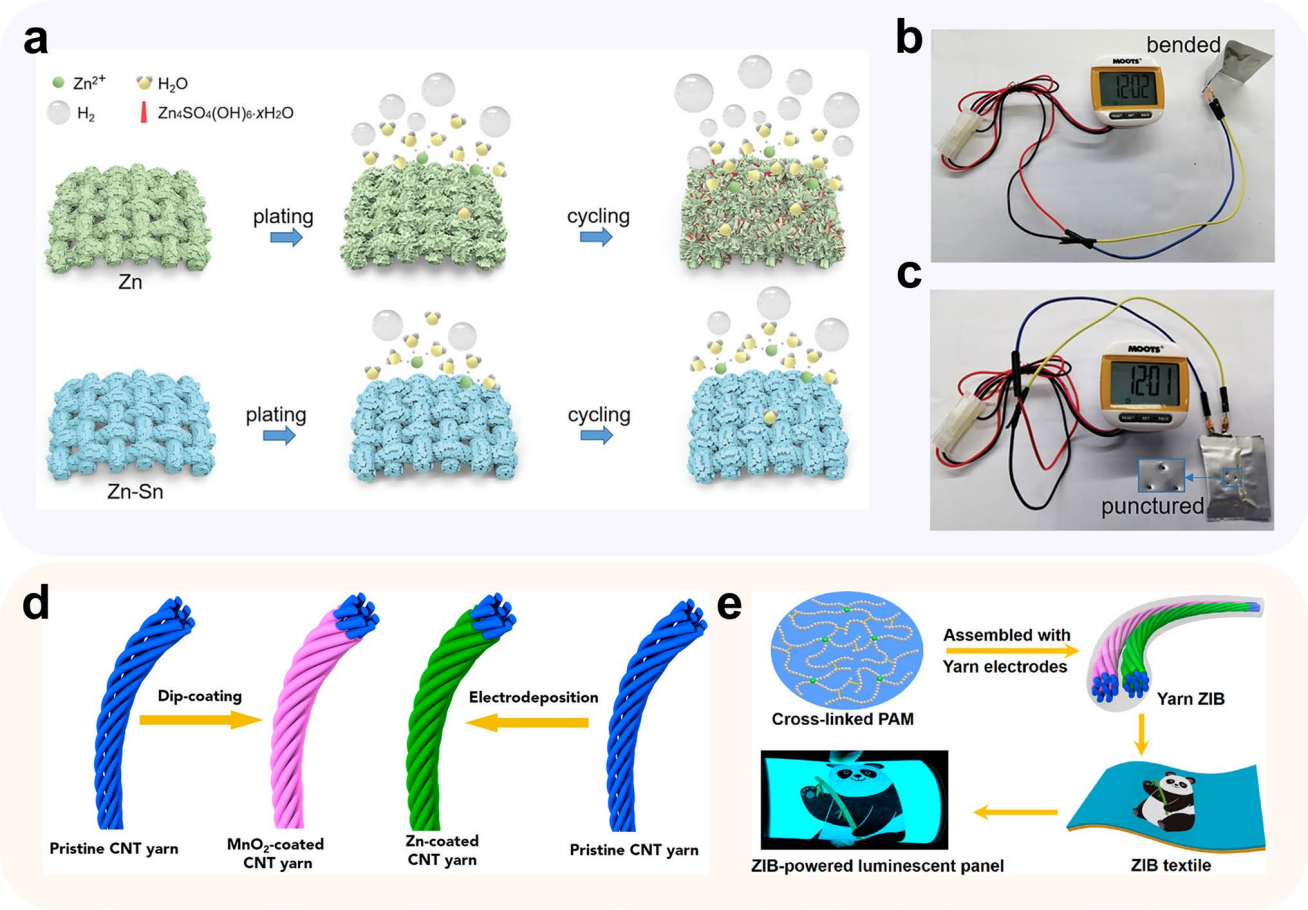
Flexible battery systems. **a** Schematic illustration of the suppressed hydrogen evolution side reactions in Zn-Sn alloy electrode, comparing with pure Zn electrode; photographs of **b** a bent ZnSn^−1^||V_2_O_5_ battery and **c** a punctured ZnSn^−1^||V_2_O_5_ battery powering a pedometer. Adapted from Ref. [146]. Copyright 2022, John Wiley and Sons; **d** schematic diagram of fabrication and encapsulation of the yarn ZIB; **e** schematic and photographs of the yarn ZIB. Adapted from Ref. [147]. Copyright 2018, American Chemical Society

The wearable and flexible batteries are believed as one of the most promising application scenarios for ZIBs, thanks to their outstanding safety and eco-friendliness. However, zinc metal, with an unsatisfying ductility, may not directly act as the anode for wearable flexible batteries [145]. Instead, the structural and material designed CCs become common candidates, modified carbon fibers in special, because of their intrinsically excellent flexibility. The sandwich-type structure is a widely used structure because of their ease of fabrication. In this structure, carbon cloth are common CCs. For example, Wang and colleagues [146] introduced Zn-Sn alloy on the surface of carbon cloth, where zinc acts as anode active material and tin offers abundant nucleation sites and inhibit the HER (Fig. 10a). Paired with the V_2_O_5_ cathode, the sandwich-type pouch cell exhibits good flexibility and safety (Fig. 10b, c). The 1D cable-type construction is another favorable structure, which is beneficial to higher volumetric energy density and multi-purpose adaption. In a typical work, Li et al. [147] designed a stretchable yarn battery using CNT as anode and cathode CCs. By roll electrodeposition and roll dip coating, Zn and MnO_2_ could be conveniently introduced on the CNT surface, and wound into a yarn battery with a quasi-solid-state flexible gel polymer electrolyte and encapsulated within a silicon tube (Fig. 10d). Subsequently, the yarn batteries are endowed with deformation robustness and shape versatility by the carbon fiber (Fig. 10e). Moreover, constructions like planar structures [148] and island-bridge structures [149] can also be considered in future applications [150].

While the structural design of 3D CCs has shown promise in improving the deposition behavior of Zn ions, the high preparation cost of most commercial or non-commercial substrates, with the exception of copper and stainless steel mesh, presents a challenge for the widespread adoption of ZIBs, let alone the increase in mass due to the complex structure of modified CCs. To advance the practicality of ZIBs, future research should not only focus on in-depth investigations into the mechanisms and modification methods of 3D CCs but also explore low-cost and high-efficiency modification means for inexpensive collectors such as copper mesh. The electrochemical performance in the Zn||CCs half-cells of some of the substrates mentioned above are summarized in Table S2.

### Crystal Orientation Preferred Materials

Zn is a hexagonal close-packed (HCP) crystal, and its various crystallographic facets exhibit differences in surface energy. The schematic diagrams of crystal planes in hexagonal Zn lattice are shown in Fig. S1. Out of the more than 20 crystalline faces of zinc, the (0 0 2) face has a dense morphology, with nine atoms coordinated to a zinc atom, as compared to six coordinated atoms in other crystalline faces. Furthermore, it exhibits the lowest surface energy, measuring approximately 0.02 eV Å^−2^ [10]. Therefore, during the electrocrystallization process of zinc, it tends to grow along the epitaxial direction (Zn < 0 0 2 >) of this face, forming dense hexagonal lamellar grains parallel to or at a smaller angle (0° to 30°) to the surface (Fig. S1a) [151]. Theoretical calculations and experiments conducted by numerous researchers have demonstrated that this low-energy crystalline surface, which is dominantly exposed during the deposition morphology of zinc according to the principles of minimum energy, can effectively suppress dendrite generation and enhance cycling life of ZIBs [152–154]. Additionally, this deposition morphology is known to suppress HER and zinc corrosion reactions that occur on zinc surface. This is due to the high hydrogen adsorption free energy and zinc loss energy possessed by this crystal surface.

However, during the actual process of zinc deposition, the dominant crystalline surface of zinc deposition is often different from the (0 0 2) surface due to several factors, such as crystal defects, electrolyte concentration gradients, local current density, substrate morphology, among others. The uncontrollable and non-directional zinc crystal plane exposure tendency may form a loose deposition morphology and generate grains vertical to the substrates (70° to 90°) (Fig. S1b, c). These factors can prove detrimental to stabilizing zinc anode [10]. Therefore, developing strategies that ensure the dominant exposure of the (0 0 2) surface becomes a promising research direction.

In contrast to the approach of constructing CCs to limit zinc dendrite growth, the crystal facet selective orientation growth strategy involves epitaxially growing atoms along the (0 0 2) crystal facet, thereby eliminating the generation of zinc dendrites at the source. This approach converts the atoms used for dendrite formation on the pristine zinc sheet surface to those grow epitaxially along the (0 0 2) crystal facet. Various methods can be introduced to achieve the deposition of the (0 0 2) dominant crystalline surface exposure of zinc, including collector modification materials [155], zinc foil pretreatment [156], artificial interfacial layers [153, 157], and electrolyte additives [158]. This section will primarily focus on the mechanism of action and the current research status of collector modification.

The zinc nucleation growth process is influenced by various factors such as the degree of crystalline surface matching of the collector, energy, and electric field distribution at the surface. As a result, the exposed crystalline surfaces during the growth process may differ from the (0 0 2) surface, and once the uniformity of this deposition behavior decreases, the risk of zinc dendrite formation will increase dramatically [41]. Hence, it is promising to modulate the (0 0 2) dominant crystallographic surface deposition of zinc by adjusting the degree of crystallographic surface matching between the collector and zinc. This degree can be characterized by the crystal lattice distortion index (*δ*), which has been extensively studied in previous research [41, 159, 160]:

9 $$\delta = \frac{{a_{\beta } - a_{\alpha } }}{{a_{\alpha } }} \times 100\%$$

where $a_{\beta }$ and $a_{\alpha }$ denote the crystal constants of the substrate and the zinc (0 0 2) crystalline surface in the stress-free state. A smaller* δ* value indicates a better match between the substrate material and the zinc (0 0 2) crystalline surface, which promotes the oriented growth of this surface. Typically, a *δ* value of less than 15% is considered an indicator of good lattice match [161]. Researchers have found that single- or few-layer graphene and its ramifications are effective materials for modifying crystallographic facet selective orientation. Additionally, the effect of single crystallographic facet-orientated metal crystals on zinc deposition morphology as a substrate has been investigated and is discussed separately in a later section.

#### Graphene Coating Layer

**Fig. 11:**
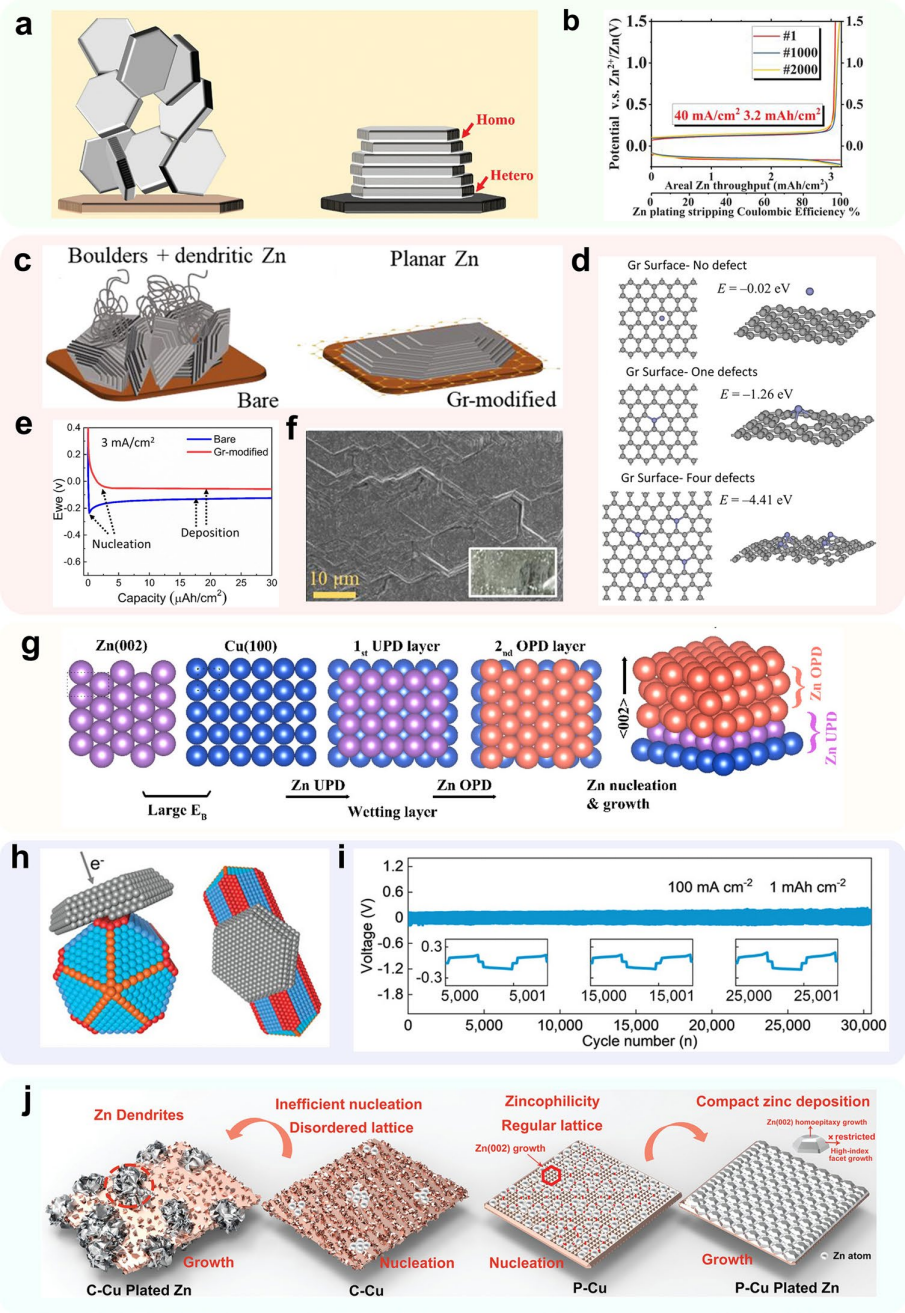
Methods using crystal orientation preferred materials. **a** Scheme illustrating the design principle of epitaxial metal electrodeposition; **b** voltage profile under high current and high areal capacity condition on GSS. Adapted from Ref. [162]. Copyright 2019, the American Association for the Advancement of Science; **c** schematic illustrating the deposition morphology of zinc on bare and graphene-modified copper foil; **d** Zn atom adsorption structures and the corresponding binding energies in the case of pristine Gr (*E* = − 0.02 eV), Gr with single defect (*E* = − 1.26 eV), and Gr with four defects (*E* = − 4.41 eV); **e** voltage–time curve during nucleation in the case of bare and graphene-modified Cu at a current density of 3 mA cm^−2^; **f** SEM imaging showing the planar nucleation of zinc deposited on graphene-modified Cu. Adapted from Ref. [163]. Copyright 2019, American Chemical Society; **g** schematic illustration of crystallographic orientation of the Zn UPD and OPD layers on Cu(100); Adapted from Ref. [164]. Copyright 2022, American Chemical Society; **h** schematic of epitaxially deposited Zn on CuNWs; **i** long-term galvanostatic deposition/dissolution of Zn@CuNWs//Zn@CuNWs symmetric cells at current density of 100.0 mA cm^−2^ with the areal capacity of 1.0 mAh cm^−2^. Adapted from Ref. [78]. Copyright 2022, John Wiley and Sons; **j** Schematic diagram of zinc deposition on different copper substrates. Adapted from Ref. [77]. Copyright 2022, John Wiley and Sons

Graphene monolayers possess a regular crystal structure and are widely utilized in various applications due to their uniform layer structure and excellent electronic conductivity [141, 146]. The monolayer crystal structure, specifically the (0 0 0 1) face of graphene, exhibits a small degree of mismatch with the (0 0 2) crystalline face of zinc (*δ* ~ 7%). This feature has been shown to be effective in creating a crystalline dominant selective modification layer (Fig. 11a). In a study by Zheng et al. [162], a method for screening crystalline dominant selective modification layers was summarized, whereby monolayer graphene was employed as a modification material. The researchers developed a fluid-based route for creating aligned graphene coatings on the substrate surface to expose and arrange graphene regularly, parallel to (0 0 0 1) crystal plane of the substrate. SEM and near-edge X-ray absorption fine structure (NEXAFS) analyses demonstrated that the zinc electrodeposited on the substrate surface grew closely epitaxially along the (0 0 2) crystal plane direction. Due to the higher free energy of hydrogen atom adsorption on the (0 0 2) crystal plane, this morphology can effectively reduce the HER reactivity and inhibit dendrite generation at the zinc anode. In half-cell tests, the electrode exhibited exceptional stability and CE, while the full cell with a high NP ratio demonstrated remarkably high capacity retention (~ 70% after 1,000 cycles at 8 mA cm^−2^ at an NP ratio of 2:1) (Fig. 11b).

However, the low hydrophilicity and zincophilicity of the graphene surface may cause polarization at the interface and increase the nucleation potential of zinc. To address this issue, Foroozan et al. [163] utilized chemical vapor deposition (CVD) to modify a graphene layer with surface defects on the surface of copper foil (Fig. 11c). Theoretical computational simulations demonstrated a significant increase in the binding energy of the graphene layer containing surface defects to zinc, from − 0.02 to − 1.26 eV (Fig. 11d). Importantly, the introduction of defects did not lead to a significant increase in the degree of lattice mismatch, which increased to only 8%. This conclusion was also confirmed by SEM analyses (Fig. 11f). Electrochemical tests confirmed the stability of this collector, with the zinc deposition overpotential of the half-cell significantly decreasing from about 100 to 30 mV at a current density of 3 mA cm^−2^, while the cycle life and CE increased steadily (Fig. 11e). Moreover, the use of doped carbon materials of other morphologies as crystallographic orientation selective materials has also been reported [165].

#### Single-Crystal Orientation Metals Coating Layer

By manipulating the crystal face orientation of metal or alloy crystals, the formation of Zn (0 0 2) crystal faces can be induced, based on the crystal face matching theory. Copper surfaces, such as (1 0 0), (1 1 0), and (1 1 1), exhibit a crystallographic mismatch with Zn (0 0 2) surface of less than 3%, indicating a high level of crystallographic selectivity [164, 166]. This crystal facet selective orientation approach holds great practical potential, as copper itself boasts excellent zincophilic and conductive properties.

In a typical example, Yan et al. [164] have probed for the mechanism of the Cu (1 0 0) plane-induced (0 0 2) epitaxially grown zinc crystal. According to the conducted theoretical simulations, the initial nucleation process of zinc atom on the (1 0 0) plane is identified as a underpotential deposition (UPD), the deposition layer of which resembles a face-centered cubic structure (FCC). The subsequent nucleation process growing upon the initial deposition layer, however, is believed an overpotential deposition, while the crystal structure translates into an HCP (Fig. 11g). To achieve uniform nucleation of Zn on the collector surface and suppress the growth of Zn dendrites, Yi et al. [78] devised a novel strategy utilizing a copper nanowire substrate with the regularly distributed Cu (1 1 1) crystalline surface (Fig. 11h). They concluded that this structure, which is similar to zinc dendrites but with controlled surface morphology, can effectively suppress the uncontrolled growth of zinc dendrites, even at higher current densities. To confirm this performance, the researchers assembled Cu NWs@Zn symmetric cells and subjected them to an ultra-high current density of 100 mA cm^−2^ with a reversible capacity of 1 mAh cm^−2^, and observed a service life of 30,000 cycles (Fig. 11i). XRD and selected area electron diffraction (SAED) verified the dominant crystalline exposure of Zn on this collector surface, while SEM confirmed the uniform distribution of zinc on the collector surface.

Despite the effectiveness of inducing crystalline surface orientation growth of zinc, the cost of such methods can be prohibitive in practical applications. To address this issue, Xie et al. [77] developed a cost-effective electrolysis method to prepare a (2 2 0) highly preferential orientation copper foil (P-Cu) by introducing a crystal facet selector. The use of P-Cu as a substrate for zinc deposition resulted in a lower nucleation overpotential and a more complete and dense hexagonal deposition morphology, as well as a higher surface zinc deposition activity and an effective crystal facet-induced orientation compared to a commercial copper foil with a random grain facet orientation (C-Cu). As deposition mass increased, Zn grew on the P-Cu surface gradually exposed two high-index crystallographic facets, (1 0 2) and (1 0 3), which promoted further deposition and cation migration (Fig. 11j). Moreover, the Zn||P-Cu half-cells exhibited better stability and average high Coulomb efficiency of 99.97%, long life of 1,100 cycles at 5 mA cm^−2^, 2 mAh cm^−2^.

Overall, crystal facet orientation preferred materials exhibit superior optimization in terms of electrochemical performance as CCs. However, challenges arise in their preparation, negating any cost advantage when compared to alternative approaches such as electrolyte additives or interfacial protective layers that yield similar effects. Nonetheless, persistent research into the mechanism and application of this strategy holds promise in mitigating its cost and operational difficulties, ultimately paving the way for the commercialization of zinc anodes [167]. The electrochemical performance in the Zn||CCs half-cells of some of the substrates mentioned above are summarized in Table S3.

## Summary and Prospect

**Fig. 12:**
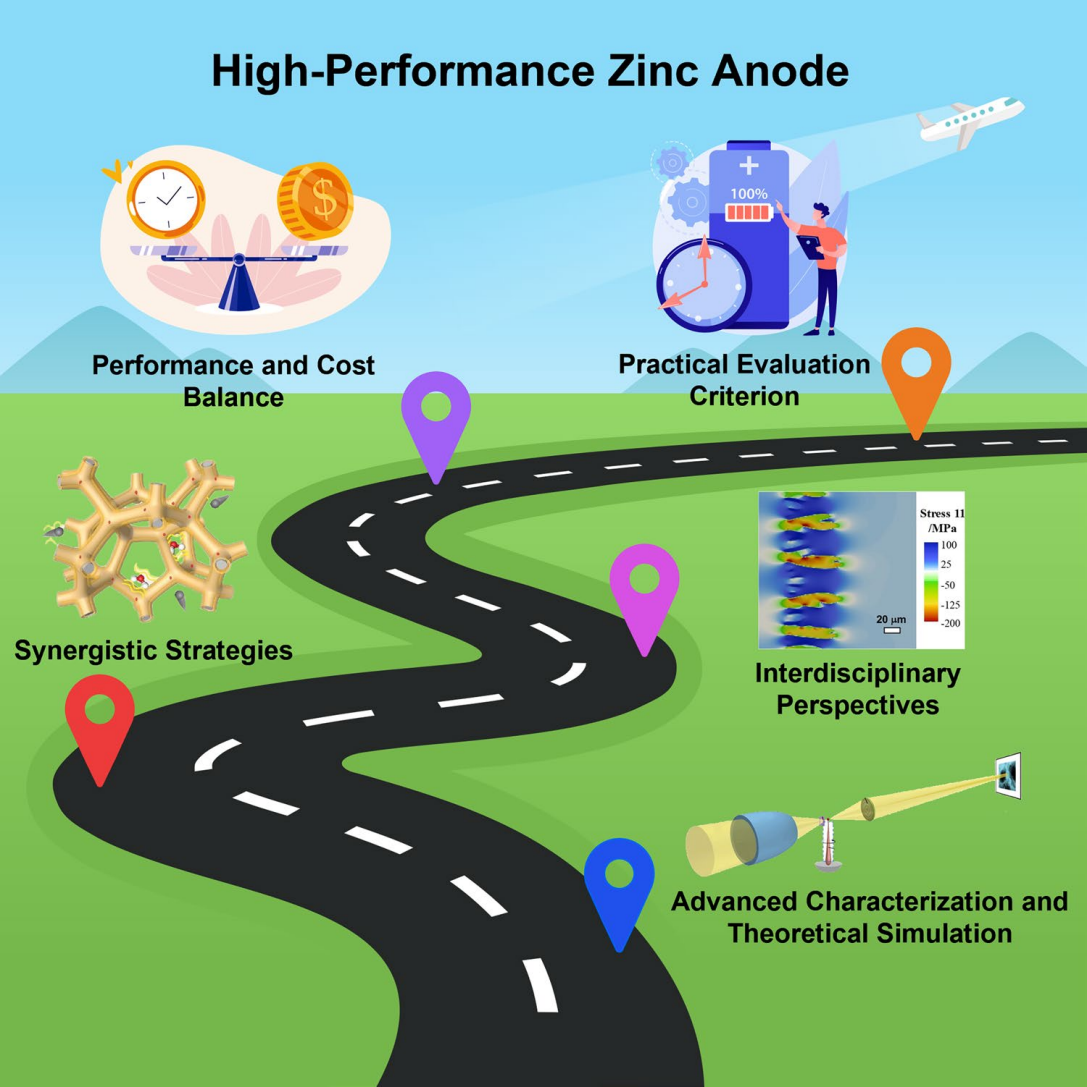
Overview of high-performance zinc anode in the present and in the future. Advanced characterization and theoretical simulation: Adapted from Ref. [168]. Open access; synergistic Strategies: Adapted from Ref. [24]. Open access; interdisciplinary perspectives: Adapted from Ref. [169]. Copyright 2020, American Chemical Society

The low-cost, safe, and environmentally friendly advantages of aqueous zinc-ion batteries have made them a rising star in the field of energy storage, despite being affected by dendrites and side reaction problems. In this review, we delve into the issues of dendrites and side reactions in zinc anodes, using conventional electrochemical dynamic theory as a basis for discussion. We also summarize various methods of introducing surface functionally and structurally designed self-supporting CCs to replace commercial zinc foils, which are classified based on their primary modification strategies. While the reviewed techniques for surface modification and structural design of zinc anode CCs have shown varying degrees of success in addressing or alleviating the challenges faced by zinc anodes, there is still room for further development in both theoretical and practical aspects. We suggest the following possible prospects for development (Fig. 12):

1. *Combination of Advanced In Situ Characterization Tools and Theoretical Simulation*: Despite extensive research, a convincing and unified model for the failure mechanism of specific battery systems in relation to dendrites and HER on zinc anode has yet to be developed. Techniques such as *in situ* Fourier transform extended-edge X-ray absorption fine structure (*in situ* FT-EXAFS), *in situ* X-ray transmission microscopy–X-ray absorption near-edge structure (*in situ* TXM-XANES), and in situ optical imaging systems can be employed alongside multi-physics field finite element simulations and molecular dynamics calculations to reveal the intrinsic causes of zinc anode failure, propose a unified mechanism, and enhance the depth of research on metal anodes.
2. *Utilization of Synergistic Strategies*: The dendritic and side reaction issues faced by zinc anodes are not independent, but rather mutually induced. While collector modifications can alleviate dendritic growth, they often fall short of fully suppressing side reactions, leading to a limited cycle life of modified zinc anodes. By combining collector modifications with strategies to suppress side reactions, such as electrolyte additives, gel electrolytes, or protective layers at the electrode/electrolyte interface, and leveraging the synergy between these approaches, researchers could simultaneously optimize zinc deposition morphology and control the involvement of active water in anode process. Thus, the electrochemical performance of zinc anodes can be further enhanced to achieve a higher stability and longer service life.
3. *Interdisciplinary Perspectives*: Despite the relatively short research history of ZIBs, thanks to the advantages of zinc anodes, the optimization of (non-rechargeable) zinc anodes and their CCs has been studied by academia and industry, even predating the research on lithium metal batteries. For instance, researchers have demonstrated the effectiveness of the tin plating strategy on the copper staple substrate of alkaline Zn||MnO_2_ cells in preventing the spontaneous corrosion of zinc, making it a potential solution for ZIBs. Furthermore, the introduction of techniques such as phase field simulations or fractal structure theory to investigate the growth process and geometry of zinc dendrites could offer new directions in the study of zinc anode mechanisms. By transferring optimization strategies from other fields to ZIBs and making targeted adjustments based on the unique characteristics of zinc anodes in ZIBs, we can explore promising avenues for zinc anode modification. Adopting an interdisciplinary approach could lead to breakthroughs in the development of highly efficient and stable zinc anodes.
4. *Balance at the Performance and Cost Levels, Respectively*: To expand the application scenarios of zinc anodes and push the boundaries of scientific research, it is crucial to continue the current cost-insensitive research on zinc anodes at the performance level. Strategies such as single-crystal plane orientation induced crystal plane epitaxial growth have shown significant potential for breaking through the bottleneck problems limiting zinc anodes. However, at the cost level, it is equally important to explore more affordable modifications and structural design methods for zinc anodes. For example, the use of biomass carbon-based collectors, copper mesh, and tin plating layer modification are promising avenues for cost reduction. Simplifying the preparation process and improving the consistency of the modification effect is also essential to compress the cost while maintaining the desired performance of the zinc anode. By balancing the performance and cost levels, researchers can advance the industrialization process of zinc anodes and make them more accessible for widespread use in energy storage applications.
5. *Practical Evaluation Criterion:* Most of the research work published in the field of zinc anode currently takes cycle life as the key performance index, which can be significantly improved by introducing excess zinc, however, the lack of standardized testing parameters for battery cycle evaluation such as current density, temperature, NP ratio, and DOD impedes the practical application of these works. Thus, the establishment of a comprehensive and standardized set of testing criteria, tailored to real-world battery usage scenarios, will provide a more reliable and relevant approach to assessing the performance of zinc anodes.

### Supplementary Information

Below is the link to the electronic supplementary material.

Supplementary file1 (PDF 322 kb)
