# Supplementary material for: Recent Advances in Structural Optimization and Surface Modification on Current Collectors for High-Performance Zinc Anode: Principles, Strategies, and Challenges
Source: Nanomicro Lett. 2023 Aug 31;15:208. doi: 10.1007/s40820-023-01177-4 (PMC10471568; doi:10.1007/s40820-023-01177-4)
Supplement: Supplementary file 1 — Supplementary file1 (PDF 322 kb) [file 40820_2023_1177_MOESM1_ESM.pdf]

Supporting Information for

Recent Advances in Structural Optimization and Surface Modification on Current Collectors for High-Performance Zinc Anode: Principles, Strategies, and Challenges

Yuxin Gong[1], Bo Wang[1,2,] *, Huaizheng Ren[1], Deyu Li[1,] *, Dianlong Wang[1], Huakun Liu[3], and Shixue Dou[3]

[1] MIIT Key Laboratory of Critical Materials Technology for New Energy Conversion and Storage, State Key Laboratory of Urban Water Resource and Environment, School of Chemistry and Chemical Engineering, Harbin Institute of Technology, Harbin 150001, Heilongjiang, P. R. China

[2] Key Laboratory of Advanced Energy Materials Chemistry (Ministry of Education), College of Chemistry, Nankai University, Tianjin 300071, P. R. China

[3] Institute of Energy Material Science, University of Shanghai for Science and Technology, Shanghai 200093, P. R. China

*Corresponding authors. E-mail: wangbo19880804@163.com (B. Wang); lideyu@hit.edu.cn (D. Li)

Supplementary Tables and Figures

Table S1 Zincophilic modification strategies for zinc anode current collectors

Materials	Optimization Strategy	Measurement Parameters (Half Cells)	Nucleation Overpotential	CE	Life Span	References
Ag@SS	Metal-based Zincophilic sites	10 mA cm^{-2} 1 mAh cm^{-2}	29 mV	99.8%	3200 cycles/640 h	[S1]
Cu NBs@NCFs	Metal-based Zincophilic sites/Structural Optimization	5 mA cm^{-2} 1 mAh cm^{-2}	63.2 mV	98.8%	1000 cycles/400 h	[S2]
CoCC	Metal-based Zincophilic Sites/Structural Optimization	20 mA cm^{-2} 1 mAh cm^{-2}	65 mV	–	800 cycles/80 h	[S3]
O, N-CC	Nonmetal-based Zincophilic Sites	1 mA cm^{-2} 1 mAh cm^{-2}	16.7 mV	98.7%	160 cycles/320 h	[S4]
Sn@NHCF	Metal-based Zincophilic Sites/Structural Optimization	5 mA cm^{-2} 5 mAh cm^{-2}	11.4 mV	99.7%	100 cycles/200 h	[S5]
Zn@SCF	Metal-based Zincophilic sites	1 mA cm^{-2} 1 mAh cm^{-2}	27 mV	98.25%	89 cycles/179 h	[S6]

Table S2 Structural optimization strategies for zinc anode current collectors

Materials	Optimization Strategy	Measurement Parameters (Half Cells)	Nucleation Overpotential	CE	Life Span	References
Ag mesh	Metal-based Zincophilic Sites/Structural Optimization	5 mA cm^{-2} 1 mAh cm^{-2}	–	99.5%	2275 cycles/910 h	[S7]
Cu Foam	Metal-based Zincophilic Materials/Structural Optimization	1 mA cm^{-2} 1 mAh cm^{-2}	65.2 mV	~98%	100 cycles/200 h	[S8]
NOCA@CF	Carbon-based Zincophilic Sites/Structural Optimization	2 mA cm^{-2} 1 mAh cm^{-2}	64 mV	95.3%	105 cycles/210 h	[S9]
Zn/CNT	Structural Optimization	2 mA cm^{-2} 2 mAh cm^{-2}<br>5 mA cm^{-2} 2.5 mAh cm^{-2}	27 mV<br>60 mV	~95%<br>97.9%	100 cycles/200 h<br>220 cycles/110 h	[S10]
3D NiZn	Structural Optimization	10 mA cm^{-2} 1 mAh cm^{-2}	–	>90%	350 cycles/70 h	[S11]
Triple-gradient Electrode	Metal-based Zincophilic Sites/Structural Optimization	10 mA cm^{-2} 1 mAh cm^{-2}	17 mV	98.7%	180 cycles/36 h	[S12]
3DP-BU@Zn	Metal-based Zincophilic Sites/Structural Optimization	10 mA cm^{-2} 1 mAh cm^{-2}	43 mV	99.9%	300 cycles/ 60 h	[S13]

Table S3 Crystal facet orientation preferred strategies for zinc anode current collectors

Materials	Optimization Strategy	Measurement Parameters (Half Cells)	Nucleation Overpotential	CE	Life Span	References
Graphene@Cu foil	Crystal Orientation preferred materials	40 mA cm^{-2} 3.2 mAh cm^{-2}	–	99.97%	10000 cycles/1600 h	[S14]
C_{flower}	Crystal Orientation preferred materials	0.5 mA cm^{-2} 0.25 mAh cm^{-2}	28.5 mV	99.3%	500 cycles/500 h	[S15]
P-Cu	Crystal Orientation preferred materials	5 mA cm^{-2} 2 mAh cm^{-2}	–	99.77%	1100 cycles/880 h	[S16]
$AgZn_3$@Zn	Metal-based Zincophilic Sites/Crystal Orientation preferred materials	1 mA cm^{-2} 1 mAh cm^{-2}	10 mV	–	375 cycles/750 h	[S17]

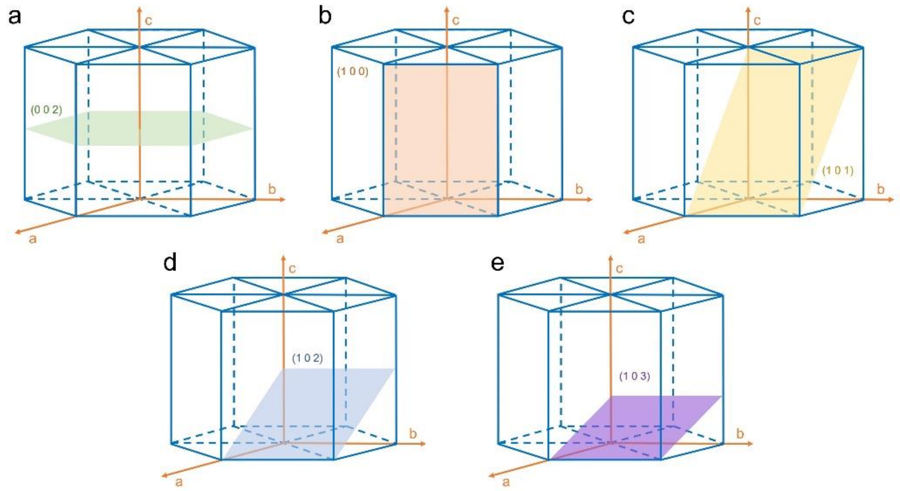

Fig. S1 Schematic diagrams of crystal planes in hexagonal Zn lattice. **a** (0 0 2) facet; **b** (1 0 0) facet; **c** (1 0 1) facet; **d** (1 0 2) facet; **e** (1 0 3) facet

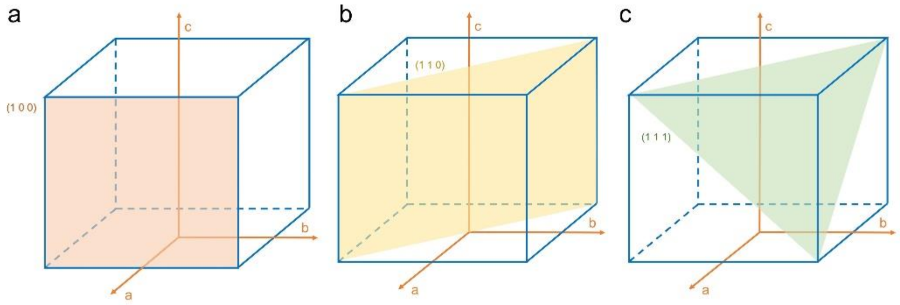

Fig. S2 Schematic diagrams of crystal planes in cubical Cu lattice. **a** (1 0 0) facet; **b** (1 0 1) facet; **c** (1 1 1) facet

Suppplementary References

[S1] Y. Zhang, G. Wang, F. Yu, G. Xu, Z. Li et al., Highly reversible and dendrite-free zn electrodeposition enabled by a thin metallic interfacial layer in aqueous batteries. Chem. Eng. J. **416**, 128062(2021). http://doi.org/10.1016/j.cej.2020.128062

[S2] Y. Zeng, P. Sun, Z. Pei, Q. Jin, X. Zhang et al., Nitrogen-doped carbon fibers embedded with zincophilic cu nanoboxes for stable zn-metal anodes. Adv. Mater. **34**(18), e2200342 (2022). http://doi.org/10.1002/adma.202200342

[S3] H. Li, C. Guo, T. Zhang, P. Xue, R. Zhao et al., Hierarchical confinement effect with zincophilic and spatial traps stabilized zn-based aqueous battery. Nano Lett. **22**(10), 4223-4231 (2022).

http://doi.org/10.1021/acs.nanolett.2c01235

[S4] M. Zhou, G. Sun, S. Zang, Uniform zinc deposition on o,n-dual functionalized carbon cloth current collector. J. Energy Chem. **69**, 76-83 (2022). http://doi.org/10.1016/j.jechem.2021.12.040

[S5] H. Yu, Y. Zeng, N. Li, D. Luan, L. Yu et al., Confining sn nanoparticles in interconnected n-doped hollow carbon spheres as hierarchical zincophilic fibers for dendrite-free zn metal anodes. Sci. Adv. **8**(10), eabm5766 (2022). http://doi.org/10.1126/sciadv.abm5766

[S6] B. Cui, Y. Gao, X. Han, W. Hu, Reversible zn stripping/plating achieved by surface thin sn layer for high-performance aqueous zinc metal batteries. J. Mater. Sci. Technol. **117**, 72-78 (2022). http://doi.org/10.1016/j.jmst.2021.10.040

[S7] R. Xue, J. Kong, Y. Wu, Y. Wang, X. Kong et al., Highly reversible zinc metal anodes enabled by a three-dimensional silver host for aqueous batteries. J. Mater. Chem. A **10**(18), 10043-10050 (2022). http://doi.org/10.1039/d2ta00326k

[S8] X. Shi, G. Xu, S. Liang, C. Li, S. Guo et al., Homogeneous deposition of zinc on three-dimensional porous copper foam as a superior zinc metal anode. ACS Sustainable Chem. Eng. **7**(21), 17737-17746 (2019). http://doi.org/10.1021/acssuschemeng.9b04085

[S9] Y. An, Y. Tian, Y. Li, C. Wei, Y. Tao et al., Heteroatom-doped 3d porous carbon architectures for highly stable aqueous zinc metal batteries and non-aqueous lithium metal batteries. Chem. Eng. J. **400**, 125843 (2020). https://doi.org/10.1016/j.cej.2020.125843

[S10] Y. Zeng, X. Zhang, R. Qin, X. Liu, P. Fang et al., Dendrite-free zinc deposition induced by multifunctional cnt frameworks for stable flexible zn-ion batteries. Adv. Mater. **31**(36), e1903675 (2019). https://doi.org/10.1002/adma.201903675

[S11] G. Zhang, X. Zhang, H. Liu, J. Li, Y. Chen et al., 3d-printed multi-channel metal lattices enabling localized electric-field redistribution for dendrite-free aqueous zn ion batteries. Adv. Energy Mater. **11**(19), 2003927 (2021). https://doi.org/10.1002/aenm.202003927

[S12] Y. Gao, Q. Cao, J. Pu, X. Zhao, G. Fu et al., Stable zn anodes with triple gradients. Adv. Mater. **35**(6), e2207573 (2023). https://doi.org/10.1002/adma.202207573

[S13] H. He, L. Zeng, D. Luo, J. He, X. Li et al., 3d printing of electron/ion-flux dual-gradient anodes for dendrite-free zinc batteries. Adv. Mater. e2211498 (2023). https://doi.org/10.1002/adma.202211498

[S14] J. Zheng, Q. Zhao, T. Tang, J. Yin, C. Quilty et al., Reversible epitaxial

electrodeposition of metals in battery anodes. Science **366**(6465), 645-648 (2019). https://doi.org/10.1126/science.aax6873

[S15] Z. Xu, S. Jin, N. Zhang, W. Deng, M. Seo et al., Efficient Zn metal anode enabled by o,n-codoped carbon microflowers. Nano Lett. **22**(3), 1350-1357 (2022). https://doi.org/10.1021/acs.nanolett.1c04709

[S16] C. Xie, H. Ji, Q. Zhang, Z. Yang, C. Hu et al., High-index zinc facet exposure induced by preferentially orientated substrate for dendrite-free zinc anode. Adv. Energy Mater. **13**(3), 2203203 (2023). https://doi.org/10.1002/aenm.202203203

[S17] H. Lu, Q. Jin, X. Jiang, Z. Dang, D. Zhang et al., Vertical crystal plane matching between AgZn(3) (002) and Zn (002) achieving a dendrite-free zinc anode. Small **18**(16), e2200131 (2022). https://doi.org/10.1002/smll.202200131
